# RNA sequencing, selection of reference genes and demonstration of feeding RNAi in *Thrips tabaci* (Lind.) (Thysanoptera: Thripidae)

**DOI:** 10.1186/s12867-019-0123-1

**Published:** 2019-02-18

**Authors:** Satnam Singh, Mridula Gupta, Suneet Pandher, Gurmeet Kaur, Neha Goel, Pankaj Rathore, Subba Reddy Palli

**Affiliations:** 1 Punjab Agricultural University, Regional Station, Faridkot, Punjab 151203 India; 2 Department of Entomology, University of Kentucky, Lexington, KY USA; 3 Forest Research Institute, Dehradun, Uttaranchal India

**Keywords:** *Thrips tabaci*, RNAseq, Reference genes, RNAi, dsRNA feeding, RNAi pathway genes

## Abstract

**Background:**

*Thrips tabaci* is a severe pest of onion and cotton. Due to lack of information on its genome or transcriptome, not much is known about this insect at the molecular level. To initiate molecular studies in this insect, RNA was sequenced; de novo transcriptome assembly and analysis were performed. The RNAseq data was used to identify reference and RNAi pathway genes in this insect. Additionally, feeding RNAi was demonstrated in *T. tabaci* for the first time.

**Results:**

From the assembled transcriptome, 27,836 coding sequence (CDS) with an average size of 1236 bp per CDS were identified. About 85.4% of CDS identified showed positive Blast hits. The homologs of most of the core RNAi machinery genes were identified in this transcriptome. To select reference genes for reverse-transcriptase real-time quantitative PCR (RT-qPCR) experiments, 14 housekeeping genes were identified in the transcriptome and their expression was analyzed by (RT-qPCR). *UbiCE* in adult, *28s* in nymphs and *SOD* under starvation stress were identified as the most stable reference genes for RT-qPCR. Feeding dsSNF7 and dsAQP caused 16.4- and 14.47-fold reduction in *SNF7* and *AQP* mRNA levels respectively, when compared to their levels in dsGFP fed control insects. Feeding dsSNF7 or dsAQP also caused 62 and 72% mortality in *T. tabaci*. Interestingly, simultaneous feeding of dsRNAs targeting *SNF7* or *AQP* and one of the RNAi pathway genes (*Dicer*-*2/Aubergine/Staufen*) resulted in a significant reduction in RNAi of target genes. These data suggest the existence of robust RNAi machinery in *T. tabaci*.

**Conclusion:**

The current research is the first report of the assembled, analyzed and annotated RNAseq resource for *T. tabaci*, which may be used for future molecular studies in this insect. Reference genes validated across stages and starvation stress provides first-hand information on stable genes in *T. tabaci.* The information on RNAi machinery genes and significant knockdown of the target gene through dsRNA feeding in synthetic diet confirms the presence of efficient RNAi in this insect. These data provide a solid foundation for further research on developing RNAi as a method to manage this pest.

**Electronic supplementary material:**

The online version of this article (10.1186/s12867-019-0123-1) contains supplementary material, which is available to authorized users.

## Background

Thrips belong to order Thysanoptera comprising of about 7400 described species [1]. These are minute insects (about 1 to 4 mm in length) with unique characteristics such as a haplodiploid genetic system, parthenogenesis, female-biased sex ratio, high fecundity, postembryonic remetaboly, asymmetrical mouthparts, short generation time, high locomotor activity and preference for concealed spaces [2, 3]. Thrips are polyphagous pests that cause economic losses to the onion, tomato, cotton, peanut, potato, pepper, corn, wheat, several ornamental plants and vegetable crops either by direct feeding or by transmitting plant viruses. The damage is caused by extensive feeding which empties the cytoplasmic and other cellular contents of plant cells [4, 5]. Due to damage perpetrated by their feeding, oviposition and ability to transmit plant viruses of genus *Orthotospovirus*, they have attained the status of a major agricultural pest globally [6–8].

Several management practices including cultural, mechanical and biological tactics are being followed worldwide to manage thrips. However, the mainstay control relies on the chemical insecticides. The development of insecticide resistance and failure of insecticides to reach the eggs in plant tissues, as well as fourth instars and pupae in the soil, hampers the effective chemical control of thrips [9]. Insecticide applications also have well-documented effects on non-target organisms and the environment. Thus, development of novel and environmentally safe control strategies are the need of the hour. RNAi, also known as PTGS (post-transcriptional gene silencing) is being developed for control of insect pests [10]. Identification of target genes for RNAi requires knowledge about genes and their expression patterns. Unfortunately, annotated and assembled transcriptome or genome sequence data are not available for *T. tabaci* in open access public databases. Therefore, RNA isolated from *T. tabaci* was sequenced, and assembled as a de novo transcriptome. We were able to retrieve *T. tabaci* homologs for most of the genes associated with RNAi response in other insects. RNAi target genes, as well as 14 genes, have also been identified and used to demonstrate RNAi and identification of stable reference genes for RT-qPCR analysis, respectively.

## Results

### De novo transcriptome

The cDNA libraries from *T. tabaci* mRNA were sequenced using Illumina paired-end sequencing technology and assembled to obtain transcriptome (Table 1). Sequencing of RNA yielded 16,737,058 (2 × 150 bp) high-quality reads. These reads were assembled into 36,342 contigs containing 200 to 16,083 nucleotides (nt) with an average length of 1816 nt per contig and the N50 of 3543. Using TransDecoder, a total of 27,836 CDS with a size between 297 and 13,161 nt and the average length of 1236 nt per CDS were identified. About 31% of the transcripts were between 200 and 500 nt in length, and a total of 8% were of size more than 5000 nt. The coding sequences predicted from these transcripts comprised of more than 31% sequences between 500 and 1000 nt and 1.43% above 5000 nt in length.

**Table 1:** Summary statistics from Illumina sequencing of *Thrips tabaci* transcriptome

Parameters	Statistics (totals)
No. of reads	16,737,058
Number of bases	4,937,528,906
Total data in Gb	4.9 Gb
No. of transcripts	36,342
Total transcript length (bases)	66,005,010
N50	3543
Maximum transcript length	16,083
Minimum transcript length	200
Mean transcript length	1816
No. of CDS	27,836
Total CDS length (bases)	34,426,740
Maximum CDS length	13,161
Minimum CDS length	297
Mean CDS length	1236
200 ≤ transcript < 500	11,320
500 ≤ transcript < 1000	6922
1000 ≤ transcript < 2000	6664
2000 ≤ transcript < 3000	3893
3000 ≤ transcript < 4000	2658
4000 ≤ transcript < 5000	1884
transcript ≥ 5000	3001
200 ≤ CDS < 500	6801
500 ≤ CDS < 1000	8534
1000 ≤ CDS < 2000	8129
2000 ≤ CDS < 3000	2562
3000 ≤ CDS < 4000	1016
4000 ≤ CDS < 5000	395
CDS ≥ 5000	399

**Fig. 1:**
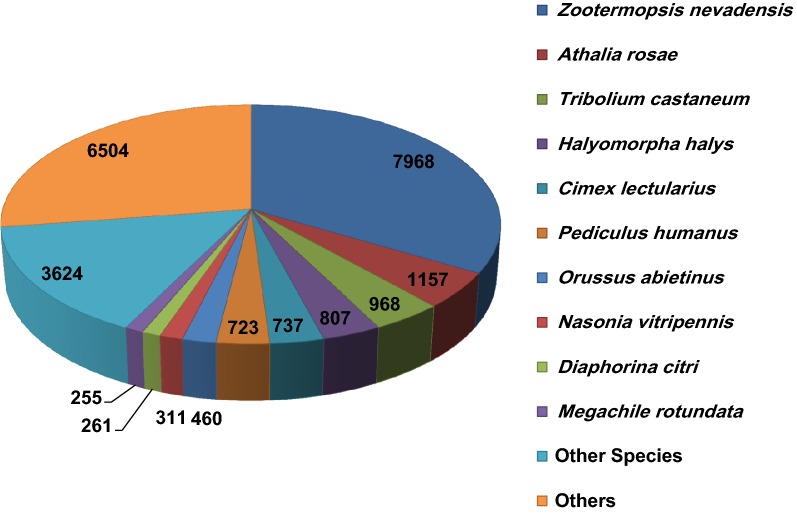
Top hits of *T. tabaci* sequences across insect and other species on the basis of highest score in BLASTX using homology searches against Nr database

For functional annotation, the assembled CDS were analyzed for gene ontology by BLASTx searches in NCBI non-redundant (nr) protein database using Blast2GO [11]. About 85.4% (23,775) of CDS showed positive blast hit matches with sequences derived from insects including *Zootermopsis nevadensis* Hagen*, Athalia rosae* Linnaeus*, Tribolium castaneum* Herbst*, Halyomorpha halys* Stål*, Cimex lectularius* Linnaeus*, Pediculus humanus* Linnaeus*, Orussus abietinus* Scopoli*, Nasonia vitripennis* Ashmead*, Diaphorina citri* Kuwayama, and *Megachile rotundata* Fabricius (Additional file 1). The predicted CDS from *T. tabaci* showed the highest similarity with *Z. nevadensis* sequences with over 7968 blast hits followed by hemipteran, *A. rosae* with over 6504 hits (Fig. 1).

**Fig. 2:**
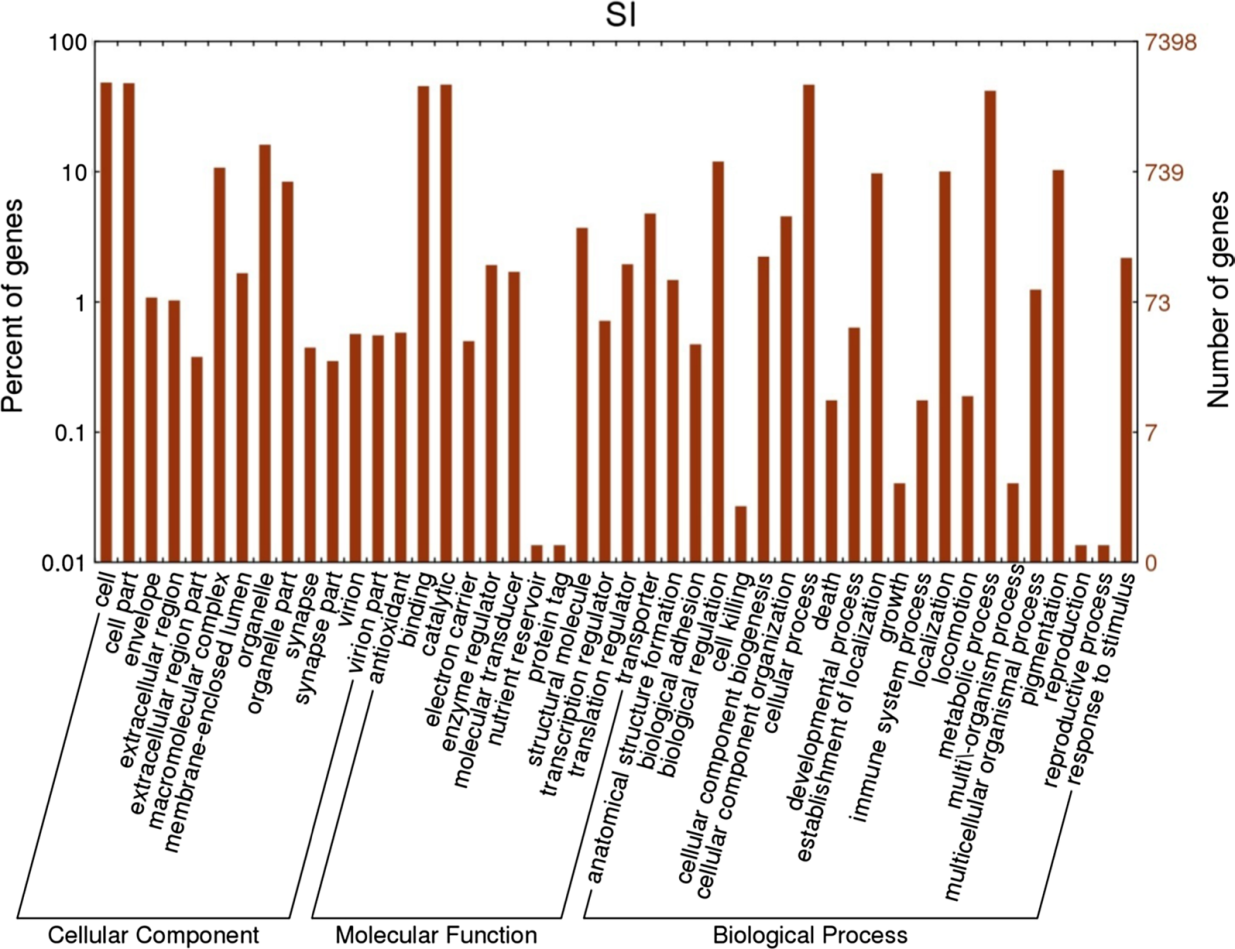
Classification of *Thrips tabaci* transcripts based on predicted gene ontology terms via WEGO plot associated with the cellular component, molecular function, and biological process. GO terms were determined using Blast2Go with an E-value cutoff of 10^−5^. In total, 7484 CDS with BLAST matches to known proteins were assigned to gene ontology

The annotation of *T. tabaci* transcriptome revealed main gene ontology (GO) categories under ‘Biological Processes’, ‘Molecular Function’ and ‘Cellular Component’ (Fig. 2). The most enriched terms for Biological processes are organic substance metabolic process (8.8%), primary metabolic process (8.3%) and cellular metabolic process (8.2%). In Molecular function, the major contributions were towards organic cyclic compound binding (7.7%) and heterocyclic compound binding (7.7%). Under Cellular component, 14.06% sequences comprised of intracellular, followed by intracellular part (11.6%), intracellular organelle (8.4%) (Additional file 2).

**Fig. 3:**
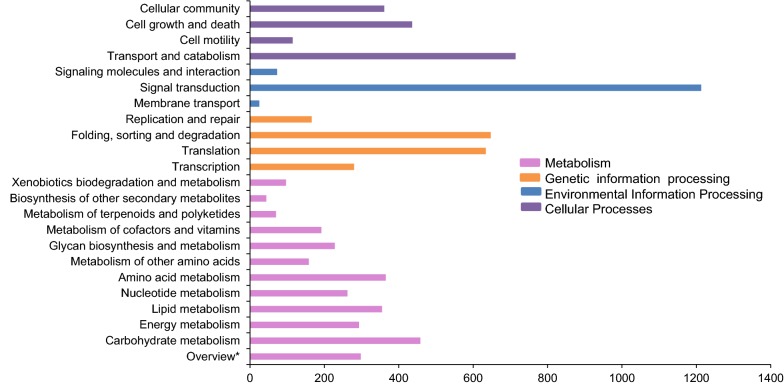
KEGG Pathway classification of CDS of *Thrips tabaci.* Overview* represents overall count for the following pathways: carbon metabolism, 2-oxocarboxylic acid metabolism Fatty acid metabolism, biosynthesis of amino acids, degradation of aromatic compounds

Functional annotation of transcripts using Kyoto Encyclopedia of Genes and Genomes (KEGG) analysis assigned KEGG Orthology (KO) IDs under four biological processes, i.e., Metabolism, Cellular, Genetic information processing and Environmental information (Fig. 3). The majority of transcripts grouped into Metabolism (37.68%) followed by Genetic information processing (23.08%). For pathways listed under this major processes, the majority of transcripts (16.02%) were associated with Signal transduction pathway, followed by Transport and catabolism (9.54%). The maximum transcripts (16.24%) under Metabolism were associated with Carbohydrate metabolism and under Genetic information processing in Folding, sorting and degradation (37.46%). Out of 27836 transcripts, only 26.88% were assigned the KO IDs using KEGG pathway analysis (Additional file 3). The completeness and quality of our assembled *T. tabaci* transcripts were evaluated by BUSCO analysis, which revealed 82.6% and 84.2% representation compared to insect and metazoan lineages, respectively [12, 13]. About 10.8% and 9.4% were “fragmented”, while 6.6% and 6.4% were missing compared to insect and metazoan lineages, respectively.

### Identification and validation of reference genes

**Fig. 4:**
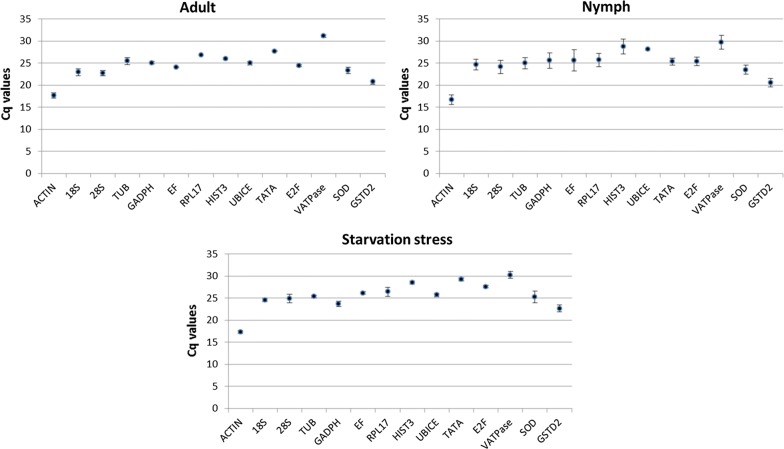
Ct values ± SD of candidate reference genes in *Thrips tabaci* nymphs, adults, and starved individuals. Each data point indicates mean ± SD of Ct values for biological triplicates

Based on published reports from other insects, fourteen candidate genes (*TUB*, *Actin*, *EF, 18s, 28s, RPL17, TATA, E2F, Hist3, UbiCE, GAPDH, vATPase, GSTD2 and SOD*) were selected for identification of stable reference genes in *T. tabaci*. The presence of selected genes in *T. tabaci* was reconfirmed by PCR amplification followed by agarose gel electrophoresis. The amplification efficiency of candidate reference genes was calculated by 3-point standard curve obtained by serial dilutions of known concentration of cDNA template. Amplification efficiency varied between 82.96 and 100.71% except for *Actin* (78.56%)*, GADPH* (72.57%)*, Hist3* (144.68%) and *vATPase* (175.1%) (Additional file 4: Table S1). The correlation coefficient values (R^2^) ranged between 0.95  and 1.00 (Table 3). Melt-curve analysis confirmed the amplification specificity, as a single peak was detected for each amplified product. The agarose gel electrophoresis of these amplified products further confirmed primer specificity through a single crisp band of the respective gene. Relative expression levels of all selected genes analyzed using RT-qPCR ranged from 17.71 (*Actin*) to 31.12 (*vATPase*) Ct value. The expression of the *vATPase* gene was considerably low (29.73–31.12) in all developmental stages tested. The Ct value of *18s, 28s, SOD* and *GSTD2* ranged between 22 and 25 across all experimental samples. However, expression of *TATA, 18s, and Hist3* showed variations among these samples (Fig. 4). The expression *EF, RPL17, TATA, E2F, Hist3, UbiCE, GAPDH, vATPase* and *Actin* was stable among insect stages and under starvation stress. However, the expression of *E2F* was substantially lower in starved animals when compared to that in normal ones.

**Fig. 5:**
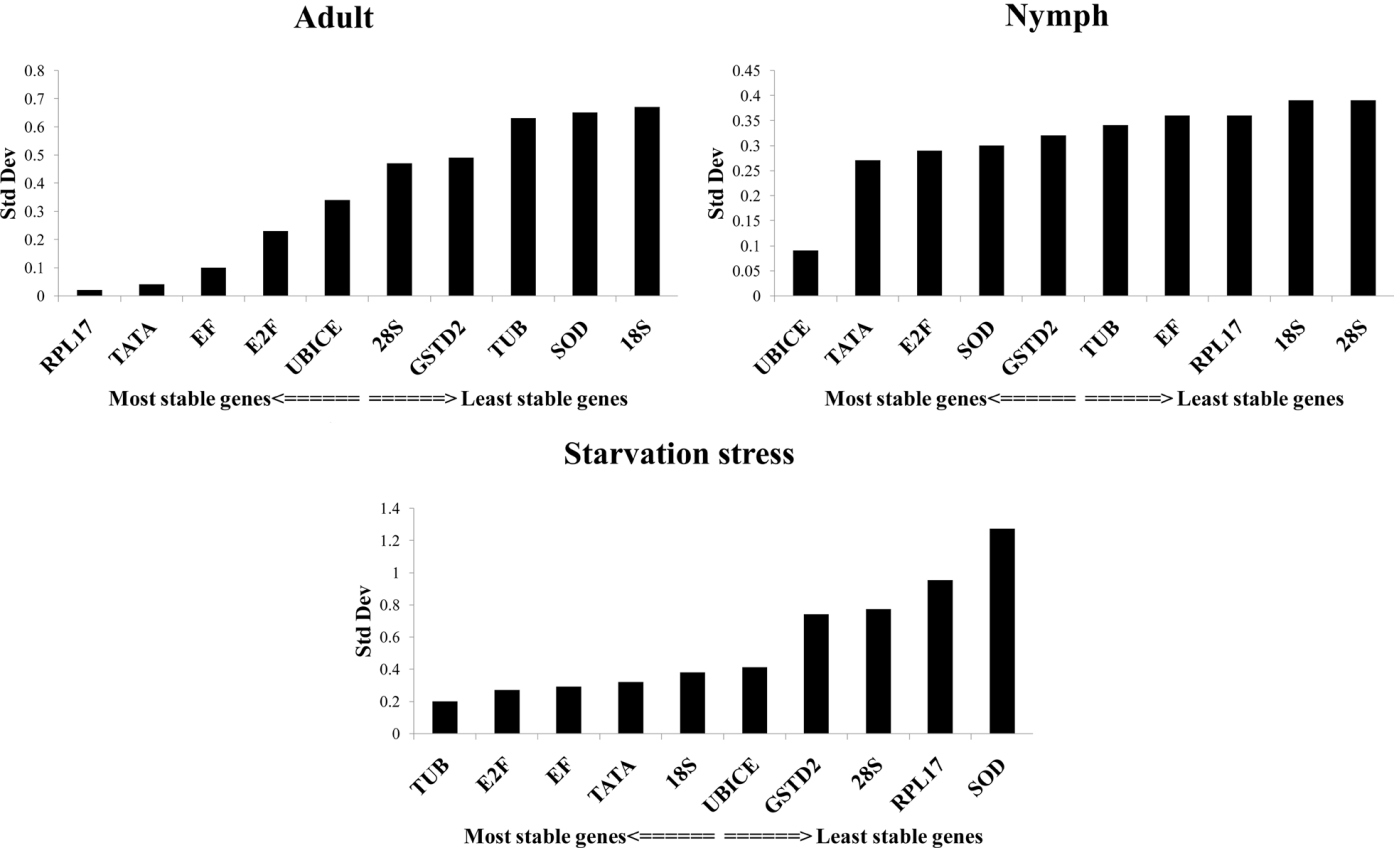
Stability analysis of candidate genes based on standard deviation of Ct values obtained by Bestkeeper analysis in *Thrips tabaci* adult, nymph, and under starvation stress

**Fig. 6:**
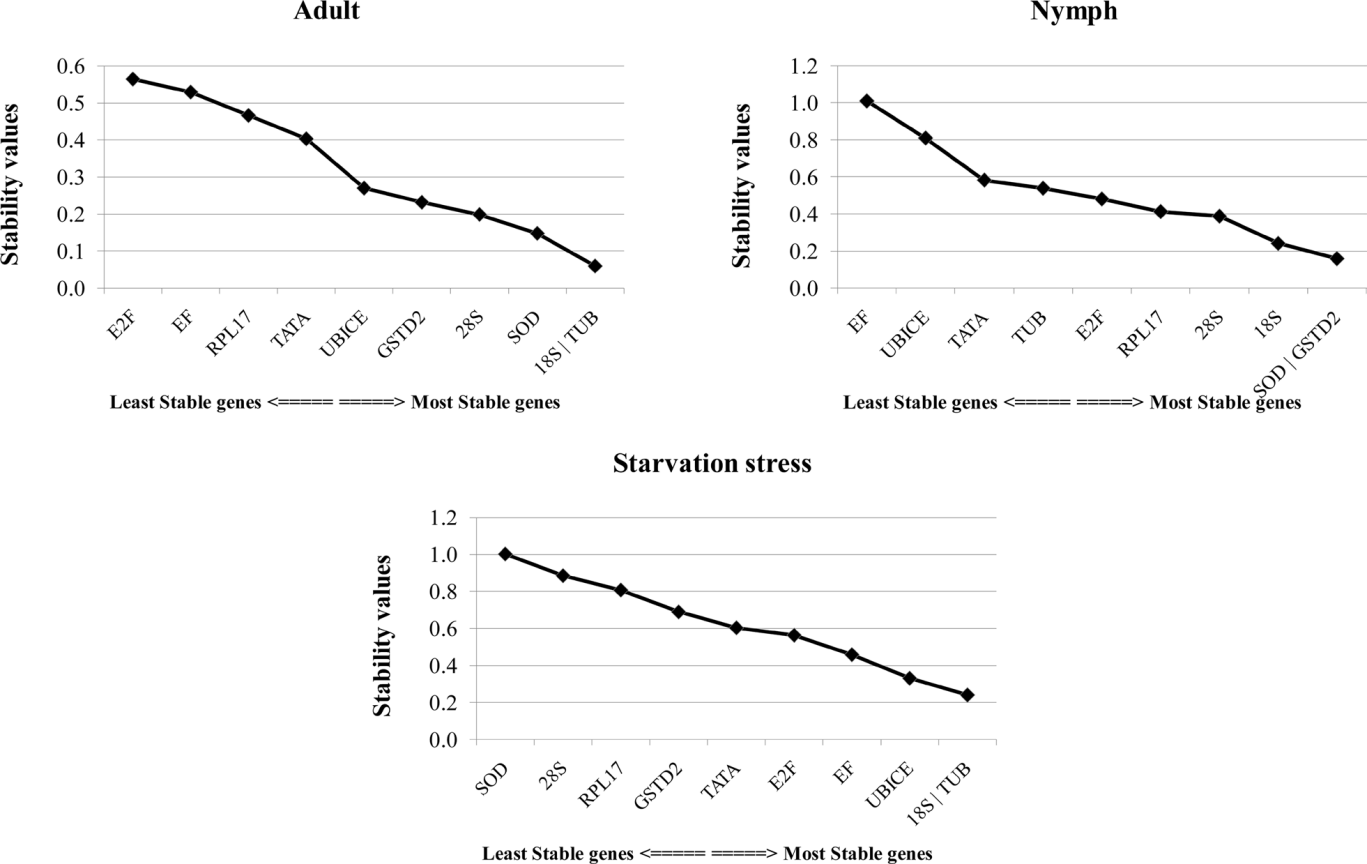
GeNorm based mean expression stability values of candidate genes in *Thrips tabaci* nymphs, adults and starved individuals

BestKeeper analysis identifies the most stable gene based on its standard deviation (SD) calculated from raw Ct values across biological replicates. SD > 1 signifies an unstable expression of the candidate gene due to high variation in its expression within a sample of the same origin. The expression of all candidate genes was stable in adult samples (SD < 1). However, in nymphs, instability was observed in the expression of *18s, 28s, TUB, EF* and *RPL17* (Additional file 4: Table S2). Based on BestKeeper analysis, *RPL17* (SD value = 0.02) in adults, *UbiCE* in nymphs (SD = 0.15) and *TUB* (SD = 0.20) under starvation stress were found to be highly stable. The candidate genes were ranked according to descending order of their stability and presented in Fig. 5. The geNorm analysis is based on two parameters, expression stability value (M) and pairwise variation (V). The highest M value indicates the least stable gene and vice versa. *SOD* and *GSTD2* (M value 0.159) in nymphs, *18s* and *TUB* in adults (M value 0.06) and under starvation stress (M value 0.24) were found to be the most stable genes (Fig. 6).

**Fig. 7:**
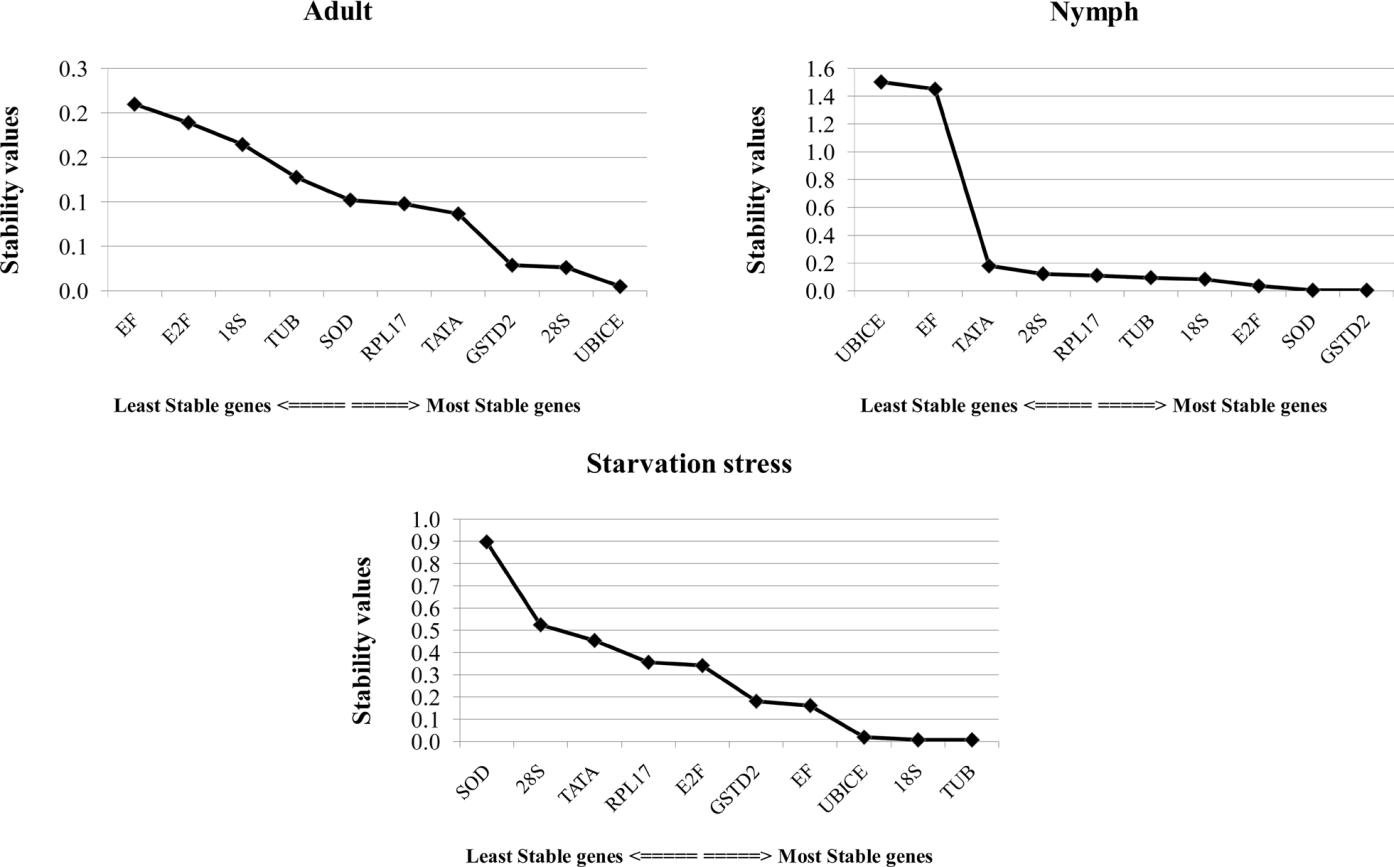
Stability of candidate genes in *Thrips tabaci* nymphs, adults and starved individuals based on NormFinder analysis

NormFinder designates the stable gene based on stability value as well as identifies the best combination of reference genes. *GSTD2* (stability value ~ 0.231) was identified as the most stable gene across all experimental samples (Additional file 4: Table S3). The overall best combination of stable genes is *18s* and *GSTD2* (stability value ~ 0.191), however, individually under starvation stress, *18s* and *TUB* were highly stable genes (stability value 0.007). Similarly, *UbiCE* (stability value—0.005) in nymphs, and *SOD* and *GSTD2* (stability value—0.003) in adults showed the highest expression. The genes ranked based on their stability values are presented in Fig. 7. Based on interpretations of both NormFinder and geNorm analysis, *18s* and *TUB* are the most stably expressed genes in starved animals. RefFinder assembles the output of BestKeeper, geNorm, and NormFinder along with Delta CT to assign an overall rank to genes based on its geometric mean (Additional file 4: Table S4 and Table 2). These analyses identified *GSTD2* and *28s* as the common stable genes.

**Table 2:** Comprehensive ranking of candidate reference genes in different stages of insect and under starvation stress using RefFinder

Adult										
Method	1	2	3	4	5	6	7	8	9	10
DELTA CT	*UbiCE*	*28s*	*GSTD2*	*TATA*	*SOD*	*RPL17*	*TUB*	*18s*	*EF*	*E2F*
BestKeeper	*RPL17*	*TATA*	*EF*	*E2F*	*UBICE*	*28s*	*GSTD2*	*TUB*	*SOD*	*18s*
Normfinder	*UbiCE*	*28s*	*GSTD2*	*TATA*	*RPL17*	*SOD*	*TUB*	*18s*	*E2F*	*EF*
Genorm	*18s|TUB*		*SOD*	*28s*	*GSTD2*	*UbiCE*	*TATA*	*RPL17*	*EF*	*E2F*
Recommended comprehensive ranking	*UbiCE*	*28s*	*TATA*	*RPL17*	*GSTD2*	*TUB*	*18s*	*SOD*	*EF*	*E2F*
Nymph										
Method	*1*	*2*	*3*	*4*	*5*	*6*	*7*	*8*	*9*	*10*
Delta CT	*SOD*	*GSTD2*	*18s*	*E2F*	*RPL17*	*28s*	*TUB*	*TATA*	*UbiCE*	*EF*
BestKeeper	*UbiCE*	*TATA*	*E2F*	*GSTD2*	*SOD*	*TUB*	*18s*	*28s*	*RPL17*	*EF*
Normfinder	*SOD*	*GSTD2*	*18s*	*E2F*	*TUB*	*RPL17*	*28s*	*TATA*	*UbiCE*	*EF*
Genorm	*SOD|GSTD2*		*18s*	*28s*	*RPL17*	*E2F*	*TUB*	*TATA*	*UbiCE*	*EF*
Recommended comprehensive ranking	*SOD*	*GSTD2*	*18s*	*E2F*	*UbiCE*	*TATA*	*28s*	*RPL17*	*TUB*	*EF*
Starvation stress										
Method	*1*	*2*	*3*	*4*	*5*	*6*	*7*	*8*	*9*	*10*
Delta CT	*TUB*	*18s*	*UbiCE*	*EF*	*GSTD2*	*E2F*	*TATA*	*RPL17*	*28s*	*SOD*
BestKeeper	*TUB*	*E2F*	*EF*	*TATA*	*18s*	*UbiCE*	*GSTD2*	*28s*	*RPL17*	*SOD*
Normfinder	*18s*	*TUB*	*UbiCE*	*EF*	*GSTD2*	*E2F*	*RPL17*	*TATA*	*28s*	*SOD*
Genorm	*18s|TUB*		*UbiCE*	*EF*	*E2F*	*TATA*	*GSTD2*	*RPL17*	*28s*	*SOD*
Recommended comprehensive ranking	*TUB*	*18s*	*UbiCE*	*EF*	*E2F*	*GSTD2*	*TATA*	*RPL17*	*28s*	*SOD*
Overall analysis										
Method	*1*	*2*	*3*	*4*	*5*	*6*	*7*	*8*	*9*	*10*
Delta CT	*GSTD2*	*18s*	*28s*	*SOD*	*TUB*	*E2F*	*EF*	*RPL17*	*TATA*	*UbiCE*
BestKeeper	*TUB*	*18s*	*RPL17*	*SOD*	*GSTD2*	*28s*	*UbiCE*	*TATA*	*E2F*	*EF*
Normfinder	*GSTD2*	*18s*	*28s*	*SOD*	*TUB*	*E2F*	*EF*	*RPL17*	*TATA*	*UbiCE*
Genorm	*SOD| GSTD2*		*18s*	*28s*	*E2F*	*EF*	*TUB*	*RPL17*	*TATA*	*UbiCE*
Recommended comprehensive ranking	*GSTD2*	*18s*	*SOD*	*TUB*	*28s*	*RPL17*	*E2F*	*EF*	*TATA*	*UbiCE*

### Impact of reference gene normalization on expression analysis in RNAi studies

**Fig. 8:**
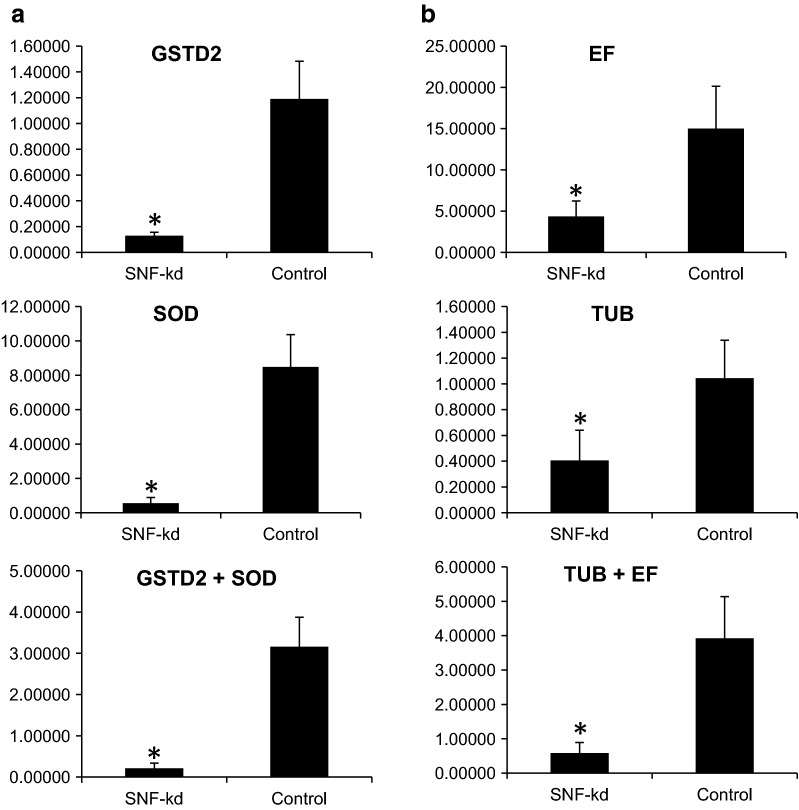
Relative expression of *SNF7* in *Thrips tabaci* after feeding dsRNA incorporated in the liquid diet through membrane feeding assay as compared control with respect to most stable and least stable reference genes. **a** Normalization studies with most stable genes (GSTD2—93.98%, SOD—93.4%, GSTD2 + SOD—93.3% Knockdown efficiency. **b** Normalization studies with least stable genes EF—70.0%, TUB—61.1%, TUB + EF—85.05% knockdown efficiency. *Indicates significant levels of mRNA transcripts of target genes compared to GFP (P ≤ 0.05, Student’s t-test)

To study the impact of identified stable reference genes on mRNA expression in RNAi experiments, the relative expression of *SNF7* gene was compared after normalization with two most and two least stable genes. The expression of *SNF7* gene was calculated in adult thrips fed with dsSNF7 after normalization with individual or combination of most stable (*GSTD2*, *SOD*) and least stable (*TUB*, *EF*) genes. The dsSNF feeding in thrips caused 93.98% and 93.4% knockdown of *SNF7* when normalized with *GSTD2* and *SOD*, respectively (Fig. 8). The use of these two genes together for normalization showed almost similar knockdown (93.3%) of *SNF7*. However, normalization with the least stable genes, i.e. *TUB* and *EF* showed 61.1% and 70.9% knockdown of *SNF7,* respectively. The normalization with individual least stable gene resulted in 22–32% less knockdown compared to normalization with stable genes. Normalization with two least stable genes (*TUB* and *EF*) showed 85.5% reduction in *SNF7* mRNA levels (Fig. 8), which was 7.8% less compared to combination of most stable genes.

### Identification of homologs of RNAi pathway genes

**Fig. 9:**
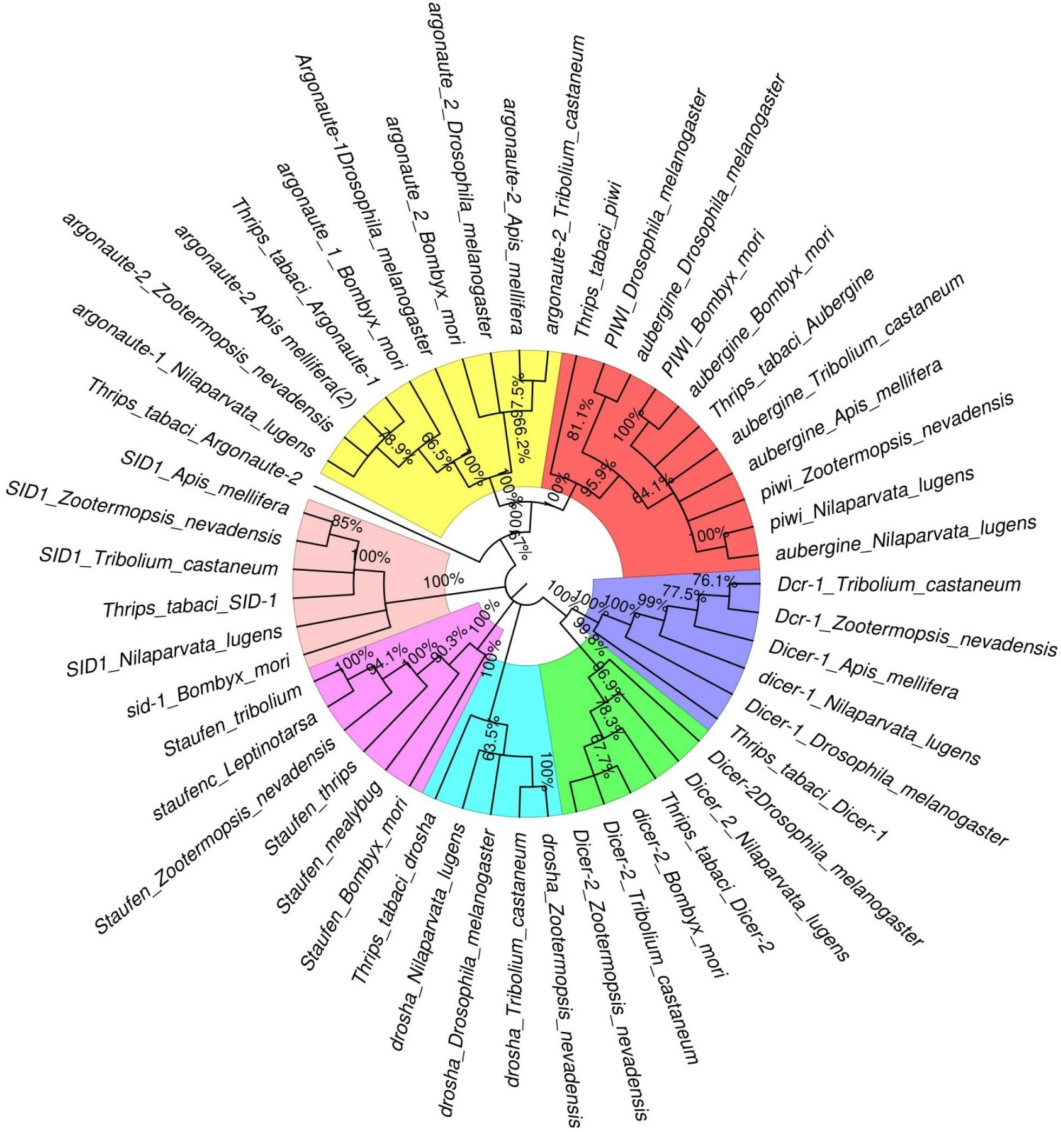
Phylogenetic tree analysis of core RNAi genes of *Thrips tabaci* with other insect groups using Neighbour-Joining method in MEGAX

**Fig. 10:**
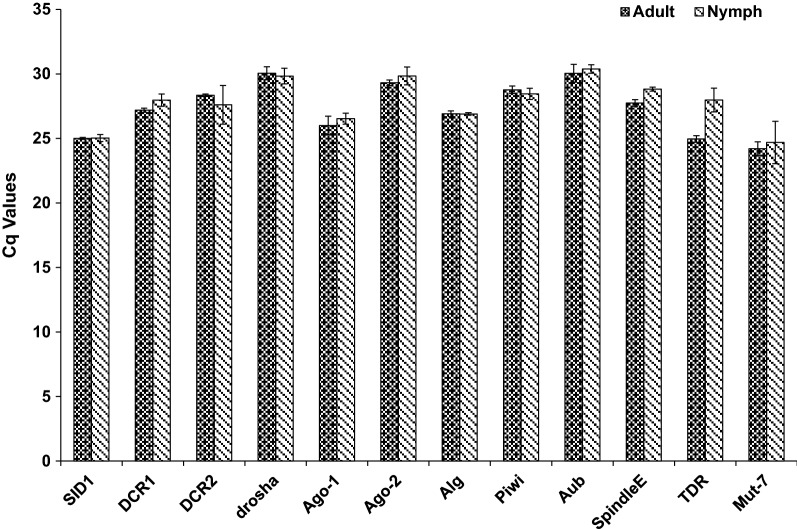
Relative expression of some genes involved in RNAi machinery in *Thrips tabaci* nymph and adult using RT-qPCR. The error bars represent the standard deviation (n = 3)

*Thrips tabaci* transcriptome was also mined to identify RNAi pathway genes. Homologs of 21 genes [*SID1, Dicer1, Dicer2, Drosha, Argonaute 1, Argonaute 2, Apoptosis linked genes (ALG), PIWI, Aubergine, abnormal spindle, Mut*-*7, HPS4, Gawky, DEAD*-*Box helicase, Tudor* (*TDR*)*, RISC, dsRNAse2, Belle, Clathrin heavy chain*, *innexin*, *Staufen* and *Clp*-1] which have been shown to function in RNAi pathway in other insects were identified in *T. tabaci* transcriptome (Additional file 4: Table S5). The homologs of genes involved in siRNA (*Argonaute 2, Dicer2),* miRNA (*Argonaute1, Dicer1*, *Drosha,*) and piRNA (*Aubergine*) pathways were identified in *T. tabaci* transcriptome. However, further analysis is required to validate their function in this insect. The core RNAi pathway genes from *T. tabaci* showed similarity with their homologs from other insect species in phylogenetic analysis (Fig. 9). The expression of 11 RNAi genes was also confirmed using RT-qPCR both in nymphs and adults of *T. tabaci* (Fig. 10). Slight variation in the expression level of these genes was observed between the life stages viz. the expression of *TDR* was high in adult compared to nymph. Within genes, the expression of *Ago*-*1, Mut*-*7* and *TDR* was higher (Cq value: 24–27) compared to other genes tested. These data provide evidence for the existence of RNAi machinery in *T. tabaci*. However, some of the genes such as *RNA*-*dependent RNA polymerase* (*RDRP*)*, Loquacious*, and *Pasha* associated with RNAi were missing in this transcriptome. This may be attributed to failure of transcriptome sequencing to include low abundant transcripts [14] or the presence of alternate proteins that can perform these functions in *T. tabaci,* which needs further investigation.

### Demonstration of feeding RNAi in *T. tabaci*

**Fig. 11:**
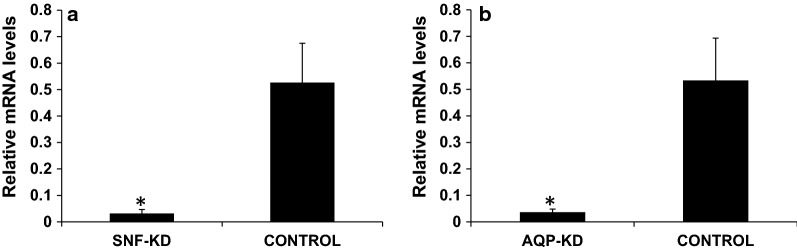
Relative expression of targeted genes in *Thrips tabaci* after feeding dsRNA incorporated in the liquid diet through membrane feeding assay. **a** *SNF7* (multivesicular protein) knockdown. **b** *AQP* (Aquaporin) knockdown. The expression level has been normalized with *GSTD2*. Error bar represents standard deviation (n = 6) and * indicates significant levels of mRNA transcripts of target genes compared to GFP (P ≤ 0.05, Student’s t-test)

To date, feeding RNAi has not been reported for any member of the insect order Thysanoptera. To confirm RNAi functioning in *T. tabaci*, 500 ng/μl of dsRNA targeting *SNF* or *AQP* gene was evaluated in membrane feeding assay. The dsRNA feeding caused 16.4- and 14.47-fold reduction in the target gene mRNA levels in dsSNF7 and dsAQP fed insects respectively, when compared to their levels in dsGFP fed control insects (Fig. 11). Feeding dsSNF7 or dsAQP also caused 62 and 72% mortality, respectively compared to 20% observed in dsGFP-fed control thrips (Fig. 14).

**Fig. 12:**
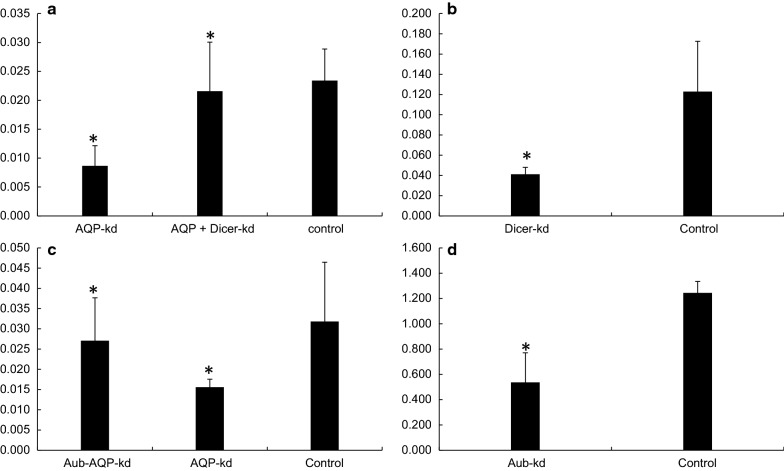
**a** RNAi of RNAi pathway genes genes in *Thrips tabaci*: **a** AQP expression levels in Dicer Knockdown. **b** Dicer Knockdown (66.7%). **c** AQP expression levels in Aubergine knockdown. **d** Aubergine knockdown (56.8%). The expression level has been normalized with *GSTD2*. Error bar represents standard deviation (n = 6) and *indicates significant levels of mRNA transcripts of target genes compared to GFP (P ≤ 0.05, Student’s t-test)

**Fig. 13:**
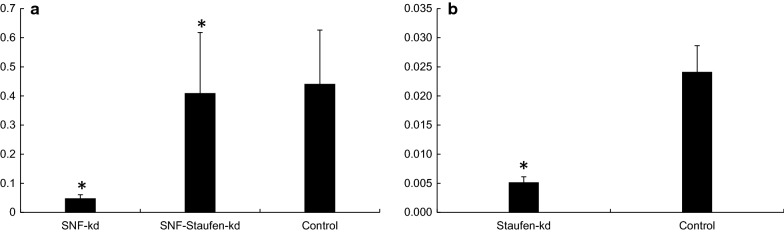
**a** SNF expression levels in *Staufen* knockown *Thrips tabaci*. **b** *Staufen* knockdown (78.5%). The expression level has been normalized with *GSTD2*. Error bar represents standard deviation (n = 6) and *indicates significant levels of mRNA transcripts of target genes compared to GFP- Control (P ≤ 0.05, Student’s t-test)

**Fig. 14:**
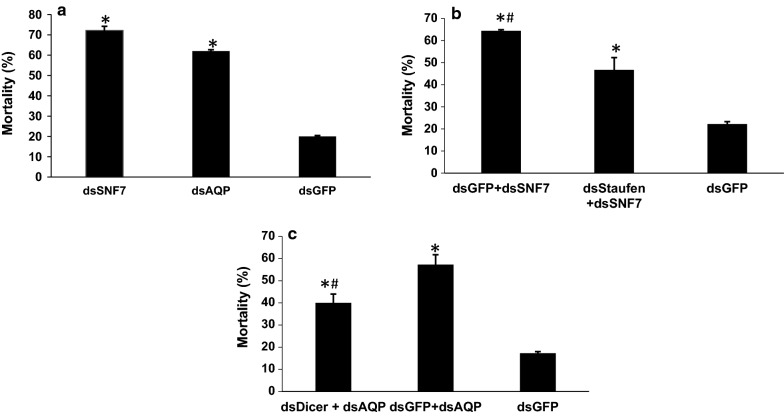
Percent mortality of *Thrips tabaci* post 48 h feeding of dsRNA incorporated in synthetic diet through membrane feeding assay. Error bars represents SE ± mean based on three replication. **a** Bioassay with dsSNF7 and dsAQP as compared to dsGFP (N = 75–100). **b** Reduction in dsSNF7 mortality due to simultaneous feeding of dsStaufen + dsSNF7 compared to dsGFP + dsSNF7 (N = 45). **c** Reduction in mortality of dsDicer + dsAQP compared to dsGFP + dsAQP (N = 75). *Indicates significant percent mortality compared to dsGFP-control and ^#^indicates significant difference between first two treatments in B and C (P ≤ 0.05, Student’s t-test)

The impact of knockdown of RNAi pathway genes on RNAi efficiency of target genes (*AQP*/*SNF7*) was evaluated through simultaneous knockdown of *Dicer*-*2, Aubergine* and *Staufen*. Feeding dsDicer-2 or, dsAubergine and dsStaufen (500 ng/ul) resulted in 78.8% (4.7-fold) and 56.8% (2.3-fold) significant reduction in the mRNA levels of target genes compared to those in dsGFP fed control insects, respectively (Figs. 12, 13). Co-administration of 500 ng/ul each of dsDicer-2 and dsAubergine in combination with 500 ng/μl dsAQP in the diet reduced the knockdown of *AQP* by 42.4 -59.8%. Feeding of a mixture of dsStaufen and dsSNF reduced 88.2% RNAi efficiency compared to dsSNF fed insects (Fig. 13). Further, the expression of *AQP* in dsDicer-2 and dsAubergine + dsAQP fed insects was 7.8–14.9% lower compared to dsGFP control, while the corresponding figure for dsStaufen + dsSNF was 7.1% (Figs. 12, 13). In addition, the feeding of dsGFP + dsSNF7 caused 64.4% mortality, which was 17.7% more compared to dsStaufen + dsSNF7. This is again indicative that knockdown of *Staufen* hindered the RNAi of *SNF7*, which in turn affected the mortality caused by dsSNF feeding in thrips Fig. 14. Similarly, simultaneous knockdown of *Dicer*-*2* and *AQP* resulted in 17.3% reduction in mortality of thrips compared to the feeding of dsGFP + dsAQP. This may also be attributed to hampering of *AQP* RNAi due to knockdown of key RNAi pathway gene i.e. *Dicer*-*2*. The results of simultaneous feeding of dsRNA against the target and RNAi pathway genes confirmed the presence of well-functioning RNAi pathway in *T. tabaci*. These data also showed that the mortality observed in dsRNA treated insects is indeed due to RNAi effect. The results have given the clue for role of *Dicer*-*2* and *Aubergine* in RNAi of *T. tabaci*. The *Thrips Staufen* analyzed for protein domains using SMART (Simple Modular Architecture Research Tool) [15] showed presence of 4 dsRBDs and one staufen domain at C-terminal, however we could find only 5 dsRBDs without Staufen C-terminal domains in insect orders other than coleoptera (Additional file 5: Fig. S1). The phylogenetic analysis revealed that the *Staufen* from *T. tabaci* shows evolutionary relationship with *Staufen* and *StauC* from coleopterans (Additional file 5: Fig. S2). The phylogenetic analysis also predicts that the *StauC* is specific only to coleopteran insects, however some unique protein motifs were predicted in *T. tabaci* using SALAD (Surveyed conserved motif ALignment diagram and the Associating Dendrogram) [16] (Additional file 5: Fig. S3).

## Discussion

The lack of genomic/transcriptomic information for Thysanoptera insect *T. tabaci* motivated us to sequence its RNA and assembly de novo transcriptome. This sequence resource was used to identify stable reference genes for RT-qPCR studies in *T. tabaci*. The data resource was also used to identify the existence of RNAi machinery and demonstration of feeding RNAi in this insect. A respectable number of CDS (27,836) successfully identified from transcripts indicated the completeness of transcriptome, which was further validated by BUSCO analysis. The quality of our assembled transcripts seems on par when compared to assemblies listed in Simao et al. [17, 18]. Previous studies with another thysanopteran insect western flower thrip, *F. occidentalis* also reported comparable number (24,262) of assembled CDS from de novo transcriptome [19]. This may be an indication of gene conservation among thysanopteran insects. The RNA-seq approach sometimes fails to include sequences of rare transcripts. However, the number of CDS identified in the current study is comparable to those reported from previously sequenced transcriptomes of other insect species including 14,797 CDS in *Drosophila melanogaster* [20]; 18,501 in *Bombyx mori* [21] and 18,071 in *Plutella xylostella* [22]. The assembled and annotated transcriptome is the first open source sequence data available for *T. tabaci*, which can be used for initiating molecular studies in this insect.

The reference gene evaluation using BestKeeper, NormFinder, geNorm and RefFinder revealed *GSTD2, UbiCE*, *28s*, *SOD* and *TUB* as stable genes. The previous reports in *Bactrocera minax* [23] and *Lucilia cuprina* [24] also showed *GSTD2* and *GST1* as the most stable reference genes, respectively. The NormFinder results, as well as overall analysis with RefFinder, showed *GSTD2* to be the most stably expressed gene in thrips nymphs and adults. Present studies also revealed *UbiCE* in adults as a highly stable gene, however overall RefFinder analysis across stages and starvation stress designated it as the least stable gene. Previous reports in different insect species with various ubiquitins, i.e. ubiquitin conjugation factor [25], polyubiquitin [26] and ubiquitin [27] have shown their suitability as a reference gene. Our previous studies in *A. biguttula* also identified *UbiCF* as a stable gene under starvation stress [28]. *SOD* gene expression levels have been found variable in *Caenorhabditis elegans* [29]. However, our studies reveal it to be the most stable gene in late nymph based on geNorm and RefFinder analysis. Thus it is clear that stability of a particular gene varies from organism to organism and even within organism under a different set of experimental conditions.

Similarly, *Actin* has been widely used as a reference gene across insect species in various expression studies [30]. However, its use as universal internal control has been questioned in many instances due to uncertainty in its expression [31, 32]. It is actually, impossible to discover a gene that is stable across different developmental stages. To reach a final decision on stable reference genes, we have done successful evaluation and validation of fourteen genes identified in the de novo transcriptome of *T. tabaci.* The results of each algorithm are variable; however, this study gives first-hand information on possible reference genes in *T. tabaci*, which may be helpful for gene expression studies to the community working on this insect. So, rather than identifying a single stable gene for each developmental stage, we have identified the top 3 to 5 genes for each developmental stage based on comprehended RefFinder analysis. Moreover, we also suggest that prior to conducting any expression analysis studies, it will be worthwhile to test and validate a few of the reported reference genes under respective experimental conditions.

To authenticate our results on reference genes and their significance in expression studies, we studied the impact of normalization with most stable (*GSTD2*, *SOD*) and least stable (*TUB*, *EF*) genes on relative expression of RNAi knockdown genes. It is evident that there was a significant difference in the knockdown percentage of *AQP* and *SNF7* when normalized individually with *GSTD2* and *SOD* in comparison to *TUB* and *EF.* For RT-qPCR studies use of two reference genes is always advisable as it ensures greater accuracy and improves the results in the expression analysis [33, 34]. We also observed that use of two reference genes significantly improves the expression results by reducing variation within the housekeeping genes. Our previous studies with RNAi in mealybug also revealed that the gene expression data normalized with unstable genes varied significantly compared to those normalized with most stable genes [28]. Thus, the use of appropriate reference genes is the key factor for interpretation of right mRNA levels in RNAi studies. The normalization with stable vs. unstable reference genes showed significant knockdown in target gene compared to control. Present study reflects that using the least stable genes for normalization may misinterpret the actual knockdown efficiency in RNAi experiments.

Transcriptome sequence resource may be helpful in generating first-hand information on genes involved in various physiological and biochemical pathways in insects. One such process, RNAi has come up as one of the important functional genomics tools and is now being exploited to identify novel targets in insects for their management [35, 36]. Sequence-specific knockdown of target genes has been so far demonstrated in many insect species [37, 38]. There is no previous report of RNAi in *T. tabaci*, so this sequence data resource revealed the presence of a good number of genes involved in RNAi. We were successful in identifying 21 RNAi pathway genes from the current transcriptome. Previous studies used the transcriptome and genome sequence data for identifying RNAi pathway genes in *Tuta absoluta* [39] and *Anoplophora glabripennis* [40] and identified a comparable number of genes associated with this pathway. These core RNAi pathways genes from *T. tabaci*, when blasted in NCBI database, showed significant similarity with their homologs from other insect species. This was further supported by the phylogenetic analysis of RNAi pathway genes from *T. tabaci* and other insect species. The phylogeny at an average bootstarp of > 60 confirmed the significant evolutionary relationship among the various gene families associated with RNAi pathway. The *SID*-*1* gene (systemic RNA interference defective-I) identified in *T. tabaci,* showed high sequence similarity with that in *Z. nevadensis*. The *SID* proteins are essential for dsRNA uptake and spreading systemic RNAi across cells [35, 41] and have been reported in many insect species [42]. Two dicer paralogues from *T. tabaci,* i.e., *Dicer*-*1* (miRNA precursors) and *Dicer*-*2* (long dsRNA endonuclease) were also identified, which are closely related to those reported from *Blattella germanica* [43]. Earlier studies in *Drosophila* suggested the role of *Dicer*-*2* in siRNA/miRNA silencing pathways as it is an important component of RISC activation complex [44]. Moreover, the knockdown of *Dicer*-*2* in *L. decemlineata* cell line partially blocked RNAi in these cells [45]. *Drosha* predicted in *T. tabaci,* is involved in the processing of miRNA precursors and has shown significant match with that of *Z. nevadensis.* However, we could not identify dsRNA binding proteins such as *RDRP* and *Loquacious* in *T. tabaci* transcriptome. The *RDRP* was also not found in *Tuta absoluta* [39]. It is possible that the expression of these genes may be induced in response to dsRNA feeding in these insects [46]. Furthermore, the homologs of *Ago*-*1, Ago*-*2, Alg*-*2, Alg*-*4, Alg*-*5, Alg*-*6, Alg*-*7, Alg*-*10, piwi, Aubergine* and *SpindleE* predicted in *T. tabaci* have shown high similarity to those reported from *Z. nevadensis* and *Locusta migratoria* [47]. The Clathrin heavy chain protein known to function in endocytosis pathway in *Leptinotarsa decemlineata* is present in *T. tabaci,* this gene exhibits high sequence similarity to that reported from *Z. nevadensis*. A single transcript *of dsRNase* has been identified from *T. tabaci. dsRNases* have been known to degrade long dsRNA in the gut of hemipteran insects, thus posing a major bottleneck in successful RNAi in this group [48, 49]. Further studies with *dsRNases* may be helpful in better understanding of RNAi in this insect. We have also identified *Staufen* in *T. tabaci*, which is a dsRNA-binding protein composed of four or five dsRBDs and initially identified from *Drosophila* [50]*. Staufen* from *C. elegans* has been known to play a role in RNAi. However recently robust RNAi efficiency in beetles has been attributed to coleopteran-specific Staufen (*StauC*), which is not present in insects of other orders [51, 52]. Thus transcriptome mining of *T. tabaci* reveals the existence of well-defined RNAi machinery in *T. tabaci*, which was further, validated through sequence-specific knockdown of *SNF7* and *AQP,* as well as core RNAi pathway genes such as *Dicer*-*2, Aubergine* and *Staufen.* The knockdown efficiency through feeding of dsSNF7 and dsAQP was about 93% in *T. tabaci*. This may be attributed to robust RNAi machinery in this insect or higher concentration of dsRNA (500 ng/µl of diet) used for feeding bioassay in the current study compared to the previous studies that reported 20 to 27% reduction in *vATPase* transcripts through injection of 80 ng dsRNA directly into the females of a thysanopteran insect, *F. occidentalis* [53]. The mortality of *T. tabaci* observed in dsSNF and dsAQP was high compared to dsGFP control and this further support the knockdown assay results of dsRNA feeding. Knockdown of RNAi pathway genes *Dicer*-*2*, *Aubergene* and *Staufen* reduced RNAi effect caused by dsSNF7 and dsAQP demonstrating the function of these genes in RNAi and cofirming that the mortality induced by dsSNF7 and dsAQP is indeed due to RNAi effect.

The simultaneous knockdown of *Dicer*-*2, Aubergine* and *Staufen* and target gene (*SNF7* and *AQP*) confirmed their role in RNAi pathway by hampering the knockdown efficiency of latter two genes. This was further supported by low mortality of *T. tabaci* observed in dsDicer + dsAQP and dsStaufen + dsSNF compared to dsAQP and dsSNF7 alone. The studies with coleopterans cell line of *L. decemlineata* showed that knockdown of *Dicer*-*2* and *Aubgerine* partially blocked RNAi in these cells [45]. Earlier studies have shown that *Staufen* in coleopterans does not play any role in RNAi, however *StauC* (*StaufenC*) predominantly contributes to this process [51]. *StauC* specific to beetles is a key player for robust RNAi response in coleopteran insects, additionally lower expression of *StauC* in RNAi resistant *L. decemlineata* cell lines (Lepd-SL1RR) showed poor RNAi response [51]. StauC domain is missing in lepidopterans which are poor processors of dsRNA to siRNA [54]. It may be possible that in other insect species like thrips, *Staufen* may be contributing to RNAi in place of *StauC*. Thrips *Staufen* have four dsRNA binding domains (dsRBD) similar to what has been reported in coleopteran specific *StauC* protein, however in most of the other insect species *Staufen* has five dsRBD [51]. The *Staufen* from *T. tabaci* was in close resemblance to coleopteran *Staufen* and additionally it showed some unique protein motifs, which were absent in other insect species. Thus *Staufen* from *T. tabaci* needs further characterization to understand its exact role in RNAi. Our studies preliminarily elucidate the role of *Dicer*-*2, Aubergine* and *Staufen* in RNAi of *T. tabaci,* which needs to be further validated through indepth studies. The validation of in silico data through mRNA expression of some predicted RNAi pathway genes, phylogenetic analysis as well dsRNA feeding assay confirm the existence of well-functioning RNAi machinery in *T. tabaci*.

## Conclusion

This study has generated good quality assembled and annotated transcriptome with 27,836 predicted CDS from *T. tabaci*. This sequence resource will be useful for molecular studies in *T. tabaci*. Fourteen putative reference genes have been identified in the transcriptome and evaluated for their stability through expression studies. Based on analysis using different algorithms, *GSTD2* has been predicted as the best reference gene in nymphs, adults and starvation stress. The top three stable genes are *UbiCE, 28s, TATA* in adults*, SOD, GSTD2 and 18s* in nymphs, and *TUB, 18s, UbiCE* under starvation conditions. Both in silico and in vivo experiments demonstrated the presence of robust RNAi machinery in *T. tabaci*. The efficient RNAi in *T. tabaci* may hold potential for developing novel strategies for the management of this pest.

## Materials and methods

### Insect culture

*Thrips tabaci* culture was maintained on *Gossypium hirsutum* variety HS 6 raised in the walk-in environmental chamber (RH 65%, Temperature 30 ± 2 °C). Staggered sowing of the cotton plants was done to ensure the year-round availability of cotton plants for the rearing of *T. tabaci*.

### Total RNA isolation and sequencing

Individuals representing each developmental stage were collected using a soft paintbrush (# 000) and pooled for total RNA isolation and transcriptome sequencing. For validation of reference genes (HKGs), a mixture of 1st and 2nd nymphal stage individuals (200 per pool) and adults (150 per pool) was collected in three replicates. In addition to this, 200 adult thrips in three replicates were kept under starvation stress for 5 h. The total RNA was isolated from these samples using Tri Reagent^**®**^ (Sigma-Aldrich) as per the manufacturer’s protocol. For RNA sequencing, the quality and quantity of the isolated RNAs were determined using denatured RNA agarose gels (Additional file 6: Fig. S1) and Qubit Fluorometer (ThermoFisher Scientific) spectrophotometer (Additional file 6: Fig. S2), respectively. For other studies the quantification of the isolated RNAs was done using Eppendorf BioSpectrometer^®^ basic and quality of RNA was checked by denaturing agarose gel electrophoresis.

RNA sequencing libraries were prepared using TruSeq stranded mRNA sample preparation kit (Illumina). mRNA was enriched from the total RNA using poly-T magnetic beads. The mRNA was fragmented enzymatically and reverse transcribed to cDNA. The cDNA was purified using XP beads (Ampure) followed by A-tailing, adapter ligation, and enrichment by PCR. The quality and quantity of the libraries were determined by 4200 Tape Station system (Agilent Technologies) using High sensitivity D1000 Screen tape (Agilent Technologies). The libraries were sequenced using the NextSeq 500 (Illumina).

### Transcriptome assembly

The sequenced raw data were processed to obtain high-quality clean reads using Trimmomatic V0.35 [55] to remove adapter sequences, ambiguous reads with unknown nucleotides “N” and more than 5% and low-quality sequences with PHRED score (QV) < 20. These high-quality reads (QV > 20), obtained were used for de novo assembly using default parameters. Adapter trimming, the Sliding window, Leading Trailing were performed at a threshold quality of below 25. Additionally, the reads were eliminated if its length was below 100 bp using Minlength setting [56]. The high-quality filtered reads were assembled into transcripts using Velvet V1.2.10 [57] and OasesV0.2.09 [58] on optimized K mer 57 and 47, respectively. To check for misassembled, erroneous and poorly supported transcripts that arose during the assembly, all high-quality reads were mapped back to their respective assembled transcripts using Burrows-Wheeler Aligner BWAV0.7.12 for validation. Open reading frames (CDS) of all assembled transcripts were predicted using TransDecoder (http://transdecoder.sourceforge.net).

### Gene ontology and functional annotation

Gene ontology and functional annotation were performed for all predicted CDS by Blast2GO program. GO mapping was carried out to retrieve GO terms for all the BLASTX functionally annotated CDS. To identify the potential involvement of the predicted CDS in biological pathways, the CDS were mapped to reference canonical pathways in KEGG. All the CDS were classified mainly into four categories: Metabolism, Cellular processes, Genetic information processing, Environmental information processing. The output of KEGG analysis included KEGG Orthology (KO) assignments, Corresponding Enzyme Commission (EC) numbers and metabolic pathways of predicted CDS using KEGG automated annotation server KASS (http://www.genome.jp/kaas-bin/kaas_main). The completeness and contiguity of the assembled transcriptome was further validated using BUSCO v1.1b1 (Benchmarking Universal Single-Copy Orthologs) 15. This analysis was performed using insect (1658) and metazoan (978) BUSCO dataset, which includes BUSCO lineages of 42 insects and 65 metazoan species.

### Identification of stable reference genes of *T. tabaci*

#### Selection of genes and primer design

Primer sequences and the associated amplicon characteristics for fourteen commonly used reference genes are summarized in Table 2. Based on reference genes described in the literature for insects [25, 28, 59], transcriptome data was searched for identification of following homologous *T. tabaci* sequences: *Actin*, *18s* (ribosomal protein 18), *28s* (ribosomal protein 28), *TUB* (tubulin), *GAPDH* (Glyceraldehyde 3-phosphate dehydrogenase), *EF* (elongation factor alpha), *RPL17* (ribosomal protein component L17), *HIST3* (Histone 3), *UbiCE* (ubiquitin-conjugating factor), *TATA* (TATA box-binding protein-like protein 1), *E2F* (transcription factor E2F1), *vATPase* (V-ATPase holoenzyme subunit B), *SOD* (Mn superoxide dismutase) and *GSTD2* (glutathione *s*-transferase D2) which were amplified using RT-qPCR (Table 3). The Primer3 software was used to design the primers [60] with predefined parameters such as amplicon length 100–150 bp, melting temperature 60–62 **°**C, primer length 20–22 bp, and GC content 45–50%.

**Table 3:** Details of candidate reference genes and primers used for RT-qPCR studies in *Thrips tabaci*

Gene symbol	Locus description	Homolog locus	Primer sequence (5′→3′)	Identity	E-value
*ACTIN*	Actin-muscle-specific	KMQ83754	Fwd: CCCTCCACCATCAAGATCAA Rev: AGATCCACATGGACTGGAA	97	7.69437E−38
*18s*	18s rRNA	XP_015126904	Fwd: CTCGAAATGCTCGAGGAAAG Rev: GAATCAGGACGTGTCTCTAACC	89	3.3679E−150
*28s*	28s ribosomal protein S29, mitochondrial	KDR19525	Fwd: GAGGGATGGGAACACATTG Rev: AAGCGCCGATCTATGTAGAAG	65	8.4952E−123
*TUB*	Tubulin gamma-2 chain	KDQ97903	Fwd: GGTTGATCCCACTCAAGTTC Rev: GGTGATTTGCGAGATAGAGC	95	0
*GADPH*	Glyceraldehyde-3-phosphate dehydrogenase 2	XP_014230283	Fwd: GAGGTTGTGTCCTCTGACTT GCCATACTCATTGTCGTACC	95	0
*EF*	transcription elongation factor SPT5, putative	XP_002428957	Fwd: GGACCTTACACTCCACAAAC Rev: GGACCCTTGATAACCTGTTG	81	0
*RPL17*	60S ribosomal protein L17	KDR19552	Fwd: CACATCGAAGTAGTGCTGAC Rev: GTTTGGCGAGCTTCTTCTTG	95	2.44944E−82
*Hist3*	Histone-lysine *N*-methyltransferase, H3 lysine-9 specific 5	KDR11973	Fwd: CAGGACAGCGAACTTATGAC Rev: CCATCTGATCCCTGATGTGT	65	0
*UbiCE*	ubiquitin conjugating enzyme	XP_954044	Fwd: ACCCAAACATAGGACTGTCG Rev: TGGATCAGCTAGGAGAGACT	53	1.31285E−26
*TATA*	TATA box-binding protein-like protein 1	KDR19315	Fwd: ACGTGGACTCAAGGATAGTG Rev: TCCCAGTCTTCATCATCTGC	88	3.6384E−116
*E2F*	Transcription factor E2F1	XP_012270447	Fwd: GCCGATTAAACCTGGAGTCT Rev: GGGCTACCATATGAGCTGTT	60	8.7238E−111
*vATPase*	V-ATPase holoenzyme subunit B	AJM13638	Fwd: CCAGGAAAGACCACTCTGAT Rev: GTCAATGCCTCTTCACCAAC	99	0
*SOD*	Mn superoxide dismutase	AIG92784	Fwd: CAAGGCAGTTGGTGTTCAAG Rev: TGCAGAGGATCTTGGTTAGC	75	5.58161E−69
*GSTD2*	Glutathione *s*-transferase D2	AFJ75818	Fwd: GTTTCAAGAGCGTCGTCAAC Rev: GACACACTCACACACACTCA	79	1.3299E−84

#### Quantitative real-time PCR analysis

Total RNA (1 µg) isolated from 2nd instar, adults and starved insects was used for cDNA synthesis using First Strand cDNA Synthesis Kit (ThermoFisher Scientific). Real-time PCR reactions were performed using three biological replicates and three technical replicates for each treatment. The reaction contained 10 µl total reaction volume (5 μl SYBR Premix Ex Taq II (Clontech Takara, USA), 1 µl (1:10) of cDNA template and 0.2 μl each of gene-specific primers (Tables 1, 2). Reactions were carried out in a 96 Lightcycler (Roche, USA) with PCR parameters as follows: 95 °C for 3 min, 40 cycles of 95 °C for 5 s, 30 s at the Tm value of primer pairs. The melting curve analysis was performed to verify the specificity of amplified PCR products.

#### Statistical analysis

To evaluate the expression stability of the reference genes in both developmental stages and starvation stress, four algorithms, geNorm [61], NormFinder [62], Bestkeeper [63] and RefFinder (http://leonxie.esy.es/RefFinder/?type=reference) were used. Ct values were obtained from the experiment run in the Lightcycler software (Roche). Comparative Ct method was used to convert these into linear values by assuming the lowest relative quantity for each gene as 1 and further using these values as input figures in NormFinder and geNorm algorithms. geNorm determines the stability score (M) by calculating the average of mean pairwise variation of each reference gene. Thus the genes with M value > 0.5 were excluded from studies. The NormFinder analysis was based on the expression stability of genes within inter and intragroup and standard deviation calculated by advanced analysis. BestKeeper is an open source MS Excel-based algorithm, which uses Ct values of genes to infer their stability taking into consideration standard deviation, P-values and correlation coefficient of each gene. Consequently, lower SD signifies the suitability of a particular gene to serve as a better internal control. RefFinder (http://leonxie.esy.es/RefFinder/?type=reference) is an online tool, which combines the outcome of all three above algorithms along with Delta CT method and calculates the geometric mean (comprehensive analysis) for each gene to rank best reference gene.

### Validation of reference genes through RNAi studies

To evaluate the impact of selection of reference genes in RNAi studies, two most stable and two least stable genes as per results obtained were used for normalization of mRNA levels in dsSNF7 (double-stranded RNA of SNF7) and dsGFP (double-stranded RNA of GFP-control) fed *T. tabaci* adults. The gene-specific primers having T7 promoter sequence (TAATACGACTCACTATAG) at 5′ end of both primers were used to amplify the template for dsSNF7 i.e. (Fwd: 5′GGTTTGCGAGGAGAGCTTATGG3′; Rev.:5′GGGAGGGCAGCAATTCCTACTT3′). The amplified product was purified using Nucleospin Gel and PCR Cleanup kit (Macherey–Nagel) as per instruction manual and used for dsRNA synthesis using MEGAscript™ RNAi Kit (Thermo Fisher Scientific). Biological samples in triplicate each comprising of 200 adults thrips were released in 1.5 ml tube (lower 1/4th part of the tube was cut and covered with 2–3 folds of muslin cloth). The dsSNF7 and dsAQP (500 ng/ul) were fed along with sucrose diet stretched between two layers of parafilm as per earlier described methodology used for insecticide bioassay [64]. Live insects were collected 48 h post-feeding, and total RNA was isolated using Tri-Reagant^®^ (Sigma-Aldrich). For relative expression studies, cDNA synthesis was done using First Strand cDNA Synthesis Kit (ThermoFisher Scientific). The cDNA from different samples was used to quantitate mRNA level of *SNF7* with RT-qPCR (as described section: Quantitative Real-time PCR analysis) using two most stable (*GSTD2* and *SOD*) and two least stable genes (*TUB* and EF) individually as well as in combination.

### Identification of RNAi pathway genes and their validation through RT-qPCR and RNAi feeding assay

To explore functional RNAi in *T. tabaci*, the sequences of major genes involved in RNAi pathway in other insects were used as query against thrips transcriptome. The E-value < e^−30^ was used as a cutoff and the identity of the retrieved sequences was confirmed through BlastX against NCBI nr database. A phylogenetic tree with the help of multiple sequence alignment was constructed using sequences of core RNAi genes identified from *T. tabaci* and their homologs reported from *D. melanogaster* M., *Bombyx mori* L., *Apis mellifera* L., *T. castaneum* Herbst, *Z. nevadensis* H. *and Nilaparvata lugens* Stål. ClustalW program and neighbor-joining analysis (bootstrap 1000) of MegaX software were used for phylogeny. To reconfirm and assess the expression levels of various RNAi pathway genes in *T. tabaci*, the primers were designed in Primer3 software and used in RT-qPCR (Table 4).

**Table 4:** Details of primers used for amplification of RNAi pathway genes

Gene name	Sequence information	Primer Name	Primer Sequence (5′→3′)
*SID*-*1*	CDS_23342_transcript_23605	qTt_SID1_F	TGTTGCTTGGTGCGATACT
		qTt_SID1_R	GGTCAGAGCTGTGTTGAGATAA
*Dicer*-*1*	CDS_24712_transcript_26352	qTt_Dcr1_F	TCTGCTTCTCGCTACGTTATG
		qTt_Dcr1_R	GTTTGCCGTTCTGCATGATAG
*Dicer*-*2*	CDS_25848_transcript_29057	qTt_Dcr2_F	GTTTGACTCAGGGAACGAAGA
		qTt_Dcr2_R	GAAGTGAGTTGACGACAGAGAG
*drosha*	CDS_15925_transcript_14592	qTt_drosha_F	TCTGCTTCTCGCTACGTTATG
		qTt_drosha_R	GTTTGCCGTTCTGCATGATAG
*Ago*-*1*	CDS_15926_transcript_14593	qTt_Ago1_F	CTCTCCCGAATTCACGACTAAC
		qTt_Ago1_R	ACTGTCTGCCTTGATCCAATAC
*Ago*-*2*	CDS_6124_transcript_5961	qTt_Ago2_F	TGGGAGAGGTTGATCCTTGTA
		qTt_Ago2_R	ACAGGTTTCTGTCGGAATATGG
*Alg*	CDS_16858_transcript_15514	qTt_Alg_F	CATCATTCCATTCCCGCTGATA
		qTt_Alg_R	CCAGAAGCATCCACTCTTGTT
*Piwi*	CDS_26052_transcript_29621	qTt_Piwi_F	CATCATTCCATTCCCGCTGATA
		qTt_Piwi_R	CCAGAAGCATCCACTCTTGTT
*Aub*	CDS_18257_transcript_16934	qTt_Aub_F	GTTCCCGACATGAACAAGAAAG
		qTt_Aub_R	AGCTCTGGCGGAAAGTATAAG
*SpindleE*	CDS_828_transcript_1424	qTt_SpindleE_F	TCGCAAGGCACTCTCTACTA
		qTt_SpindleE_R	CAGAGGACAGCGTTGATTGT
*Tdr*	CDS_9987_transcript_9161	qTt_Tdr_F	GTCATCACACCAGATCCTAACC
		qTt_Tdr_R	GGATACTGCGAGTTACCTCTTC
*Mut*-*7*	CDS_3222_transcript_3490	qTt_Mut-7_F	TTATCTGTGCCGGTGGAATAC
		qTt_Mut-7_R	CCAAGCTACGATACCCTCTTG

We chose *SNF7* and *AQP* (Aquaporin) as target genes to confirm the RNAi functioning in *T. tabaci*. *SNF7* functions as a constituent of *ESCRT* (Endosomal Sorting Complex Required for Transport) pathway, which is responsible for the cellular functions such as internalization, transport, sorting and lysosomal degradation of transmembrane proteins [65]. Aquaporins are majorly involved in water regulation in some insect species such as phloem-feeding leafhopper *Cicadella viridis* [66] and *B. tabaci* [67]. Gene-specific primers to amplify fragments of *SNF7* (Fwd. 5′TAATACGACTCACTATAGGGTTTGCGAGGAGAGCTTATGG3′; Rev.:5′TAATACGACTCACTATAGGGGAGGGCAGCAATTCCTACTT3′) and *AQP* (Fwd: 5′TAATACGACTCACTATAGGGGAGATGAAGTACACGATGGC3′; Rev.: 5′TAATACGACTCACTATAGGGCGCAGCACATCTGGATAA3′) with T7 promoter sequence at 5′ ends of both forward and reverse primer were custom synthesized. The product amplified from these primers was purified using Nucleospin Gel and PCR Cleanup kit (Macherey–Nagel) as per instruction manual. The purified product was used as template for the dsRNA synthesis using MEGAscript™ RNAi Kit (Thermo Fisher Scientific). Biological samples in triplicate each comprising of 200 insects both nymph and adult thrips were released in 1.5 ml tube (lower 1/4th part of the tube was cut and covered with 2–3 folds of muslin cloth) (Additional file 6: Fig. S3). The dsSNF7 and dsAQP (500 ng/μl) were fed along with sucrose diet stretched between two layers of parafilm as per earlier described methodology used for insecticide bioassay [64]. Similarly synthesized dsRNA against *Dicer*-*2, Aubergine* and *Staufen* genes was evaluated to examine their respective knockdown impact on RNAi of *AQP*/*SNF7* gene (Additional file 4: Table S6). For this, 500 ng/μl each of dsDicer-2 and dsAubergine mixed with dsAQP (500 ng/μl) while dsStaufen mixed with dsSNF was co-administered to thrips in sucrose diet separately for the each RNAi pathway and target gene. Live insects were collected 48 h post-feeding, and total RNA was isolated using Tri-Reagant^®^ (Sigma-Aldrich). cDNA was synthesized from 1 µg total RNA using First Strand cDNA Synthesis Kit (ThermoFisher Scientific) followed by RT-qPCR analysis as described in the earlier section (Quantitative Real-time PCR analysis). In a separate experiment, a bioassay was conducted in three replicates (70–100 adults in each) to assess the mortality of thrips after feeding 500 ng/µl of dsSNF, dsAQP, dsDicer-2 + dsAQP, dsAubergine + dsAQP and dsStaufen + dsSNF7 and compared with dsGFP control. Further, *Staufen* sequences were analyzed through SMART and SALAD to study conserved domains and motifs, respectively [15, 16].

## Additional files

**Additional file 1.** Annotations of *T. tabaci* CDS sequences.

**Additional file 2.** Gene ontology.

**Additional file 3.** KEGG Automatic Annotation Server (KASS) pathway analysis.

**Additional file 4.** Reference gene amplicon characteristics, analysis with NormFinder, BestKeeper and RefFinder, list of RNAi pathway genes identified from *T. tabaci* and dsRNA primer details.

**Additional file 5.** Staufen protein domains from different insect, phylogenetic analysis of Staufen from insect species and Unique motifs in *T. tabaci* Staufen compared to other insects.

**Additional file 6.** RNA quality assessment for transcriptome sequencing and dsRNA feeding schematic layout.

## Abbreviations

RT-qPCR: real-time quantitative PCR
RNA: ribonucleic acid
dsRNA: double stranded ribonucleic acid
RNAi: RNA interference
NCBI: National Center for Biotechnology Information
TSA: Transcriptome Shotgun Assembly
SRA: Sequence Read Archives
KASS: KEGG Automatic Annotation Server
KEGG: Kyoto Encyclopedia of Genes and Genomes
cDNA: complementary deoxy-ribonucleic acid
CDS: coding sequence
RNA-seq: RNA sequencing
GO: gene ontology
BUSCO: Benchmarking Universal Single-Copy Orthologs
18S: ribosomal protein 18
28s: ribosomal protein 28
*TUB*: tubulin
*GAPDH*: glyceraldehyde 3-phosphate dehydrogenase
*EF*: elongation factor alpha
*RPL17*: ribosomal protein component L17
*HIST3*: histone 3
*UbiCE*: ubiquitin-conjugating factor
*TATA*: TATA box-binding protein-like protein 1
*E2F*: transcription factor E2F1
*VATPase*: V-ATPase holoenzyme subunit B
*SOD*: Mn superoxide dismutase
*GSTD2*: glutathione *s*-transferase D2
GFP: green florescent protein
Ct: cycle threshold
SD: standard deviation
