# Supplementary material for: RNA sequencing, selection of reference genes and demonstration of feeding RNAi in Thrips tabaci (Lind.) (Thysanoptera: Thripidae)
Source: BMC Mol Biol. 2019 Feb 18;20:6. doi: 10.1186/s12867-019-0123-1 (PMC6380046; doi:10.1186/s12867-019-0123-1)
Supplement: Supplementary file 4 — Additional file 4. Reference gene amplicon characteristics, analysis with NormFinder, BestKeeper and RefFinder, list of RNAi pathway genes identified from T. tabaci and dsRNA primer details. [file 12867_2019_123_MOESM4_ESM.pdf]

Table S1: Characteristics of amplicons of candidate reference genes amplified using gene specific primers

Gene Symbol	Product Tm (°C)	Amplicon length	Efficiency	Amplification efficiency	Correlation coefficient
Actin	84	110	1.79	78.56	0.99
18s	82	100	1.85	84.58	0.97
28s	79	100	1.88	88.3	0.99
TUB	82	114	1.83	82.96	0.98
GADPH	82	120	1.73	72.57	0.94
EF	84	111	2.01	100.71	1
RPL17	83	103	1.95	94.97	0.95
Hist3	82	113	2.45	144.68	0.99
UbiCE	83	113	1.87	86.8	0.99
TATA	81	114	1.97	97.3	1
E2F	83	100	1.92	92.11	0.98
vATPase	81	103	2.75	175.1	0.79
SOD	84	102	1.96	95.94	0.99
GSTD2	80	100	1.86	86.38	0.99

Table S2. Detailed Bestkeeper analysis for all candidate reference genes

ADULT											
		**18S**	**28S**	**TUB**	**EF**	**RPL17**	**UBICE**	**TATA**	**E2F**	**SOD**	**GSTD2**
**geo mean**	**CP**	22.93	22.75	25.46	24.09	26.85	24.99	27.74	24.38	23.31	20.76
**AR mean**	**CP**	22.94	22.76	25.47	24.09	26.85	24.99	27.74	24.38	23.32	20.77
**Min**	**CP**	21.94	22.05	24.53	23.94	26.82	24.48	27.68	24.15	22.35	20.17
**Max**	**CP**	23.94	23.47	26.41	24.24	26.88	25.50	27.79	24.72	24.10	21.51
**Stddev**	**[± CP]**	0.67	0.47	0.63	0.10	0.02	0.34	0.04	0.23	0.65	0.49
**Cv**	**%CP**	2.91	2.08	2.46	0.42	0.07	1.36	0.13	0.93	2.77	2.38
**Min**	**x-fold**	-1.98	-1.63	-1.90	-1.11	-1.02	-1.42	-1.04	-1.17	-1.94	-1.51
**Max**	**x-fold**	2.02	1.64	1.93	1.11	1.02	1.43	1.04	1.27	1.73	1.68
**Stddev**	**[± x-fold]**	1.59	1.39	1.54	1.07	1.01	1.27	1.02	1.17	1.57	1.41
**Pearson correlation coefficient**											
**BestKeeper vs.coeff. of correlation**		0.997	0.997	0.997	0.001	0.997	0.997	0.997	0.001	0.994	0.969
**p-value**		0.001	0.001	0.001	0.001	0.001	0.001	0.001	0.837	0.001	0.007
**NYMPH**											
		**18S**	**28S**	**TUB**	**EF**	**RPL17**	**UBICE**	**TATA**	**E2F**	**SOD**	**GSTD2**
**geo Mean [CP]**	**CP**	24.66	24.13	24.97	25.53	25.67	28.13	25.33	25.37	23.50	20.56
**ar Mean [CP]**	**CP**	24.69	24.18	25.00	25.65	25.71	28.13	25.34	25.39	23.52	20.58
**min [CP]**	**CP**	23.84	23.09	23.46	23.24	24.49	27.91	24.41	24.28	22.53	19.77
**max [CP]**	**CP**	26.36	26.30	26.54	29.00	27.87	28.24	26.27	26.64	25.00	22.00
**stddev [± CP]**	**[± CP]**	1.12	1.41	1.03	2.24	1.44	0.15	0.62	0.83	0.98	0.94
**CV [% CP]**	**%CP**	4.52	5.85	4.12	8.72	5.59	0.52	2.46	3.27	4.18	4.59
**min [x-fold]**	**x-fold**	-1.76	-2.06	-2.85	-4.90	-2.26	-1.16	-1.89	-2.14	-1.96	-1.73
**max [x-fold]**	**x-fold**	3.25	4.49	2.97	11.05	4.60	1.08	1.92	2.40	2.83	2.71
**stddev [± x-fold]**	**[± x-**	2.17	2.66	2.04	4.71	2.71	1.11	1.54	1.78	1.98	1.92

	**fold]**										
**Pearson correlation coefficient (r)**											
**BestKeeper vs.coeff. of corr. [r]**		0.912	0.933	0.924	0.96	0.976	0.001	0.956	0.971	0.999	0.969
**p-value**		0.031	0.021	0.025	0.01	0.004	0.028	0.011	0.006	0.001	0.006
**STARVATION STRESS**											
**GENE**		**18S**	**28S**	**TUB**	**EF**	**RPL17**	**UBICE**	**TATA**	**E2F**	**SOD**	**GSTD2**
**geo mean**	**CP**	24.57	24.90	25.33	26.16	26.41	25.72	29.21	27.59	25.24	22.64
**AR mean**	**CP**	24.57	24.92	25.33	26.16	26.43	25.72	29.21	27.59	25.27	22.65
**Min**	**CP**	24.00	23.77	25.03	25.92	25.00	25.40	28.87	27.28	24.00	22.06
**Max**	**CP**	24.97	26.06	25.63	26.59	27.39	26.34	29.69	28.00	27.18	23.76
**Stddev**	**[± CP]**	0.38	0.77	0.20	0.29	0.95	0.41	0.32	0.27	1.27	0.74
**Cv**	**%CP**	1.56	3.08	0.79	1.10	3.61	1.61	1.10	0.99	5.03	3.27
**Min**	**x-fold**	-1.48	-2.19	-1.23	-1.18	-2.66	-1.25	-1.26	-1.24	-2.36	-1.49
**Max**	**x-fold**	1.32	2.23	1.23	1.35	1.97	1.54	1.40	1.33	3.85	2.18
**Stddev**	**[± x-fold]**	1.30	1.70	1.15	1.22	1.94	1.33	1.25	1.21	2.41	1.67
**Pearson correlation coefficient**											
**BestKeeper vs.coeff. of correlation**		0.968	0.549	0.999	0.117	0.962	0.83	0.001	0.001	0.928	0.859
**p-value**		0.007	0.338	0.001	0.851	0.009	0.082	0.006	0.166	0.023	0.062

Table S3 : Normfinder based stability values of different candidate genes

GENES	ADULT	NYMPH	Starvation Stress
18s	0.164	0.083	0.007
28s	0.026	0.121	0.525
TUB	0.128	0.094	0.007
EF	0.210	1.451	0.162
RPL17	0.098	0.109	0.357
UbiCE	0.005	1.503	0.019
TATA	0.086	0.178	0.455
E2F	0.189	0.034	0.342
SOD	0.102	0.003	0.899
GSTD2	0.029	0.003	0.181

Table S4 : Geometric mean ranking of candidate reference genes using RefFinder

**ADULT**		**NYMPH**		**Starvation stress**	
**GENES**	**Geomean of ranking values**	**GENES**	**Geomean of ranking values**	**GENES**	**Geomean of ranking values**
UbiCE	2.34	*E2F*	2.06	*TUB*	1.19
28s	3.13	*SOD*	2.11	*18s*	1.78
TATA	3.87	*TATA*	2.51	*UbiCE*	3.57
RPL17	3.94	*GSTD2*	3.66	*EF*	3.72
GSTD2	4.21	*UbiCE*	5.62	*E2F*	4.36
TUB	4.45	*TUB*	5.63	*GSTD2*	5.92
18s	5.03	*EF*	5.63	*TATA*	6.05
SOD	5.33	*RPL17*	5.79	*RPL17*	7.97
EF	7.02	*28s*	8.18	*28s*	8.74
E2F	7.75	*18s*	9	*SOD*	10

Table S5 :Listof genes associated with RNAi mechanism identified in the *T. tabaci* assembled transcriptome at *E*-value < e^{-30}. *Similarity; #Alignment length

Gene	CDS/Transcript Number	Annotation	S-value	S*	AL#
**DsRNA uptake**					
SID-1	CDS_15961_transcript_14627<br>CDS_15962_transcript_14627<br>CDS_15963_transcript_14628	gi|646715429|gb|KDR19041.1|SID1 transmembrane family member 1 [*Zootermopsisnevadensis*]	5.09814E-49<br>0	77<br>70<br>72	178<br>622<br>798
Clathrin Heavy Chain	CDS_194_transcript_841<br>CDS_24400_transcript_25704<br>CDS_25967_transcript_29416<br>CDS_25967_transcript_29416	gi|646720852|gb|KDR22435.1|Clathrin heavy chain [*Zootermopsisnevadensis*]	0<br>1.13E-77<br>1.1E-178<br>1.1E-178	97<br>96<br>94<br>94	1676<br>137<br>323<br>323
Innexin2	CDS_13894_transcript_12670<br>CDS_4215_transcript_4271<br>CDS_13893_transcript_12669<br>CDS_13894_transcript_12670	gi|242014052|ref|XP_002427712.1|Innexin inx2, putative [*Pediculushumanuscorporis*]	0<br>0<br>0<br>0	86<br>91<br>86<br>86	399<br>358<br>399<br>399
**dsRNA cleavage**					
Dicer-1	CDS_24712_transcript_26352<br>CDS_25848_transcript_29057<br>CDS_23342_transcript_23605	gi|240247227|emb|CAX68236.1|dicer-1 [*Blattellagermanica*]	0<br>5.3605E-161<br>1.38053E-30	64<br>78<br>71	807<br>415<br>154
Dicer-2	CDS_20968_transcript_20034<br>CDS_21294_transcript_20494	gi|406034947|emb|CCF23094.1|Dicer 2 [*Blattellagermanica*]	3.9936E-157<br>0	47<br>56	1709<br>1653
Drosha	CDS_20526_transcript_19507	gi|646694518|gb|KDR08017.1|Ribonuclease 3 [*Zootermopsisnevadensis*]	0	86	982
**Endonuclease activity**					
Argonaute 1	CDS_15926_transcript_14593<br>CDS_15921_transcript_14590<br>CDS_15922_transcript_14590<br>CDS_15923_transcript_14591	gi|516306731|gb|AGO85968.1|argonaute 1, partial [*Locustamigratoria*]	0<br>0<br>0<br>0	99<br>97<br>97<br>97	403<br>855<br>418<br>855

Argonaute 2	CDS_6124_transcript_5961<br>CDS_6122_transcript_5961<br>CDS_6123_transcript_5961<br>CDS_6125_transcript_5962<br>CDS_6126_transcript_5962<br>CDS_6128_transcript_5963<br>CDS_6130_transcript_5965<br>CDS_18274_transcript_16947<br>CDS_25525_transcript_28247	gi|723784142|gb|AIY24303.1|Argonaute 2 [*Laodelphaxstriatella*]	0<br>1.06074E-90<br>6.0224E-160<br>0<br>0<br>0<br>0	98<br>75<br>64<br>67<br>67<br>67<br>67	433<br>223<br>583<br>818<br>818<br>818<br>818
PCDG 6 (ALG-6)	CDS_16858_transcript_15514<br>CDS_24257_transcript_25393	gi|646723721|gb|KDR24234.1|Programmed cell death protein 6 [*Zootermopsisnevadensis*]	5.7885E-114<br>5.7885E-114	97<br>97	175<br>175
PIWI	CDS_11559_transcript_10537<br>CDS_11560_transcript_10538	gi|646703429|gb|KDR12118.1|Piwi-like protein 1 [*Zootermopsisnevadensis*]	0<br>0	74<br>75	782<br>773
Aub	CDS_26052_transcript_29621<br>CDS_24257_transcript_25393	gi|817196653|ref|XP_012273923.1|PREDICTED: protein aubergine-like isoform X2 [*Orussusabietinus*]	8.02156E-39<br>8.49505E-40	64<br>77	148<br>109
Asp Spindle E	CDS_828_transcript_1424<br>CDS_829_transcript_1425	gi|646720534|gb|KDR22214.1|Protein abnormal spindle [*Zootermopsisnevadensis*]	3.87747E-39<br>9.26456E-68	50<br>73	558<br>232
**Others**					

Tudor	CDS_9987_transcript_9161	gi|646703152|gb|KDR11968.1|Tudor	2.95608E-80	58	139
	CDS_9990_transcript_9164	domain-containing protein 5	1.13593E-11	55	182
	CDS_10607_transcript_9637	[*Zootermopsisnevadensis*]	1.2331E-12	52	182
	CDS_10608_transcript_9638		1.20765E-12	52	182
	CDS_11871_transcript_10813		1.20765E-12	52	435
	CDS_11872_transcript_10813		1.9006E-123	66	435
	CDS_11873_transcript_10813		1.9006E-123	66	90
	CDS_11874_transcript_10814		9.21698E-09	53	188
	CDS_11875_transcript_10815		5.36742E-16	54	622
	CDS_11876_transcript_10815		1.56031E-87	53	188
	CDS_11877_transcript_10815		5.36742E-16	54	90
	CDS_11878_transcript_10816		9.21698E-09	53	188
	CDS_11879_transcript_10816		5.36742E-16	54	622
	CDS_11880_transcript_10816		1.56031E-87	53	90
	CDS_12288_transcript_11176		9.21698E-09	53	622
	CDS_14189_transcript_12964		1.56031E-87	53	188
	CDS_14190_transcript_12965		5.36742E-16	54	39
	CDS_15383_transcript_14106		2.65212E-07	69	1183
	CDS_15384_transcript_14107		4.8541E-153	50	163
	CDS_15385_transcript_14108		4.9947E-14	53	85
	CDS_15386_transcript_14109		1.21319E-07	54	832
	CDS_15387_transcript_14109		8.90154E-90	50	827
	CDS_15388_transcript_14110		2.1975E-90	50	85
	CDS_15389_transcript_14111		1.08125E-07	54	827
	CDS_15390_transcript_14111		2.1975E-90	50	832
	CDS_17731_transcript_16404		7.85433E-90	50	181
	CDS_17732_transcript_16405		3.02975E-30	61	599
	CDS_22526_transcript_22311		7.68318E-60	49	569
	CDS_23795_transcript_24504		1.52443E-60	49	570
	CDS_24864_transcript_26674		2.4689E-61	49	513
	CDS_3219_transcript_3489		2.17002E-24	44	469
	CDS_3220_transcript_3489		1.08973E-30	46	285
	CDS_3221_transcript_3489		2.58141E-26	48	

Mut-7	CDS_3222_transcript_3490<br>CDS_3223_transcript_3490<br>CDS_3224_transcript_3490<br>CDS_3225_transcript_3491<br>CDS_3226_transcript_3491<br>CDS_3227_transcript_3492<br>CDS_3228_transcript_3492<br>CDS_3229_transcript_3492<br>CDS_3230_transcript_3493<br>CDS_3231_transcript_3493<br>CDS_3232_transcript_3494<br>CDS_3233_transcript_3494<br>CDS_3234_transcript_3494	gi|646704864|gb|KDR12833.1|putative exonuclease mut-7-like protein [*Zootermopsisnevadensis*]	5.4797E-153<br>3.36557E-10<br>6.96901E-13<br>5.4797E-153<br>6.96901E-13<br>3.36557E-10<br>2.61635E-30<br>5.4797E-153<br>3.36557E-10<br>6.96901E-13<br>5.4797E-153<br>3.0543E-172<br>3.36557E-10	63<br>64<br>61<br>63<br>61<br>64<br>54<br>63<br>64<br>61<br>63<br>59<br>64	579<br>62<br>98<br>579<br>98<br>62<br>222<br>579<br>62<br>98<br>579<br>728<br>62
HPS4	CDS_18050_transcript_16726<br>CDS_18051_transcript_16727	gi|646704291|gb|KDR12556.1|Hermansky-Pudlak syndrome 4 protein [*Zootermopsisnevadensis*]	0<br>0	57<br>57	929<br>929
Belle	CDS_2216_transcript_2724<br>CDS_3872_transcript_4046	gi|805814300|ref|XP_012148170.1|PREDICTED: DEAD-box ATP-dependent RNA helicase 20 [*Megachilerotundata*]	4.07E-08<br>0	66<br>87	57<br>481
Gawky	CDS_1202_transcript_1771<br>CDS_1203_transcript_1772<br>CDS_1204_transcript_1773<br>CDS_1199_transcript_1770<br>CDS_1207_transcript_1774<br>CDS_1209_transcript_1775	gi|572313755|ref|XP_006622570.1|PREDICTED: protein Gawky-like [*Apisdorsata*]	2.41E-51<br>2.41E-51<br>2.41E-51<br>2.41E-51<br>2.41E-51<br>2.41E-51	68<br>68<br>68<br>68<br>68<br>68	187<br>187<br>187<br>187<br>187<br>187
CLP1	CDS_17314_transcript_15991	gi|646701253|gb|KDR11058.1|CLP1-like protein [*Zootermopsisnevadensis*]	0	88	414
V-type proton ATPase subunit H	CDS_9906_transcript_9079<br>CDS_9907_transcript_9080<br>CDS_9908_transcript_9080<br>CDS_9909_transcript_9081<br>CDS_9910_transcript_9082	gi|646704147|gb|KDR12485.1|V-type proton ATPase subunit H [Zootermopsisnevadensis]	0<br>1.65118E-96<br>8.4095E-179<br>8.4095E-179<br>0	80<br>85<br>92<br>92<br>88	558<br>182<br>289<br>289<br>484

<table>
<tr><td>Rab-7a</td><td>CDS_24868_transcript_26695
CDS_24869_transcript_26696
CDS_1706_transcript_2230
CDS_1707_transcript_2231
CDS_1708_transcript_2232
CDS_21319_transcript_20533
CDS_21320_transcript_20534</td><td>>gi|936578738|ref|XP_014204028.1|PREDICTED: ras-related protein Rab-7a [Copidosomafloridanum]
>gi|936578741|ref|XP_014204029.1| PREDICTED: ras-related protein Rab-7a [Copidosomafloridanum]
>gi|936578744|ref|XP_014204030.1| PREDICTED: ras-related protein Rab-7a [Copidosomafloridanum]
>gi|936578747|ref|XP_014204031.1| PREDICTED: ras-related protein Rab-7a [Copidosomafloridanum]
>gi|936578750|ref|XP_014204032.1| PREDICTED: ras-related protein Rab-7a [Copidosomafloridanum]
>gi|936578753|ref|XP_014204033.1| PREDICTED: ras-related protein Rab-7a [Copidosomafloridanum]
>gi|936578756|ref|XP_014204034.1| PREDICTED: ras-related protein Rab-7a [Copidosomafloridanum]
>gi|936578759|ref|XP_014204035.1| PREDICTED: ras-related protein Rab-7a [Copidosomafloridanum]
>gi|936578762|ref|XP_014204037.1| PREDICTED: ras-related protein Rab-7a [Copidosomafloridanum]
>gi|936578765|ref|XP_014204038.1| PREDICTED: ras-related protein Rab-7a [Copidosomafloridanum]</td><td>9.8E-128
1.1E-127
7E-133
7E-133
7E-133
4.5E-113
3.3E-115</td><td>93
93
93
93
93
87
72</td><td>208
208
206
206
206
207
287</td></tr>
<tr><td>RISC</td><td>CDS_2188_transcript_2700
CDS_4702_transcript_4696
CDS_2185_transcript_2699</td><td>gi|646700117|gb|KDR10415.1|putative RISC-loading complex subunit [Zootermopsisnevadensis]</td><td>6.2E-23
3E-149
6.2E-23</td><td>48
81
48</td><td>353
331
353</td></tr>
</table>

DEAD box protein	CDS_17843_transcript_16505<br>CDS_3872_transcript_4046<br>CDS_19922_transcript_18854<br>CDS_2216_transcript_2724	gi|242021722|ref|XP_002431292.1|DEAD box ATP-dependent RNA helicase, putative [Pediculushumanuscorporis]<br>>gi|212516560|gb|EEB18554.1| DEAD box ATP-dependent RNA helicase, putative [Pediculushumanuscorporis]	5.8E-110<br>0<br>4.99E-06<br>4.07E-08	61<br>87<br>65<br>66	474<br>481<br>84<br>57
dsRNAse 2	CDS_24848_transcript_26641	gi|599126388|gb|AHN55089.1|dsRNase 2, partial [Schistocercagregaria]	2.93687E-36	76	111
RDRP	CDS_2003_transcript_2493<br>CDS_2006_transcript_2493<br>CDS_2007_transcript_2494<br>CDS_2009_transcript_2494<br>CDS_2010_transcript_2494<br>CDS_2012_transcript_2495<br>CDS_2013_transcript_2495<br>CDS_2017_transcript_2495<br>CDS_2018_transcript_2496<br>CDS_2020_transcript_2496<br>CDS_2021_transcript_2496<br>CDS_2023_transcript_2497<br>CDS_2024_transcript_2497<br>CDS_2026_transcript_2498<br>CDS_2027_transcript_2498<br>CDS_2028_transcript_2498<br>CDS_2030_transcript_2498<br>CDS_2032_transcript_2499	gi|527355229|gb|AGS13094.1|RdRP [Cowpea mild mottle virus]	0<br>2.3133E-06<br>9.2932E-174<br>0<br>0<br>4.7604E-153<br>9.2932E-174<br>0<br>0<br>9.2932E-174<br>0<br>9.2932E-174<br>2.3721E-156<br>0<br>0<br>2.22675E-31<br>9.2932E-174<br>0	95<br>44<br>87<br>95<br>57<br>95<br>87<br>57<br>95<br>87<br>57<br>87<br>56<br>95<br>74<br>45<br>87<br>57	330<br>198<br>331<br>330<br>1027<br>255<br>331<br>1027<br>330<br>331<br>1027<br>331<br>833<br>330<br>512<br>477<br>331<br>1027

Table S6: Primer sequences for dsRNA template of potential RNAi machinery

**Genes**	**dsRNA Primer Sequence**
Dicer-2	Fwd: 5'TAATACGACTCACTATAGGGCCATCACCTACTCATTC3'<br>Rev: 5'TAATACGACTCACTATAGGGGTTAGTAGAGCCTTCAG3'
Staufen	Fwd: 5'TAATACGACTCACTATAGCAGGAGCCTCTTTATAC 3'<br>Rev: 5'TAATACGACTCACTATAGCCTTAGGAAAGTCTGAG3'
Aubergine	Fwd: 5'TAATACGACTCACTATAGGGCTAAAGCTGAGAAGAGG3'<br>Rev: 5'TAATACGACTCACTATAGGGCACCTCCGTATTTACTC3'
