# Supplementary material for: RNA sequencing, selection of reference genes and demonstration of feeding RNAi in Thrips tabaci (Lind.) (Thysanoptera: Thripidae)
Source: BMC Mol Biol. 2019 Feb 18;20:6. doi: 10.1186/s12867-019-0123-1 (PMC6380046; doi:10.1186/s12867-019-0123-1)
Supplement: Supplementary file 5 — Additional file 5. Staufen protein domains from different insect, phylogenetic analysis of Staufen from insect species and Unique motifs in T. tabaci Staufen compared to other insects. [file 12867_2019_123_MOESM5_ESM.pdf]

Fig. S1 TheStaufen sequences and their domains characterized across different insect species using SMART (Simple Modular Architecture Research Tool)

Thripstabaci

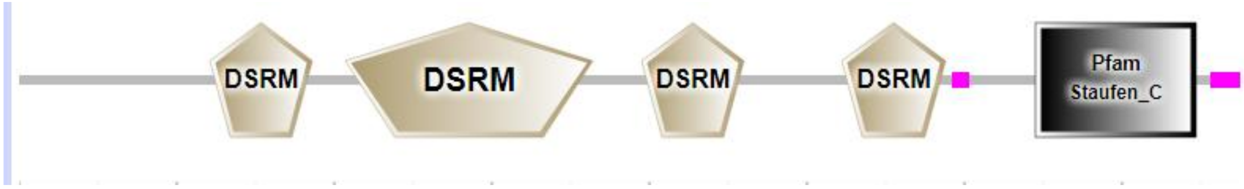

*Frankliniellaoccidentalis*XM_026420079.1

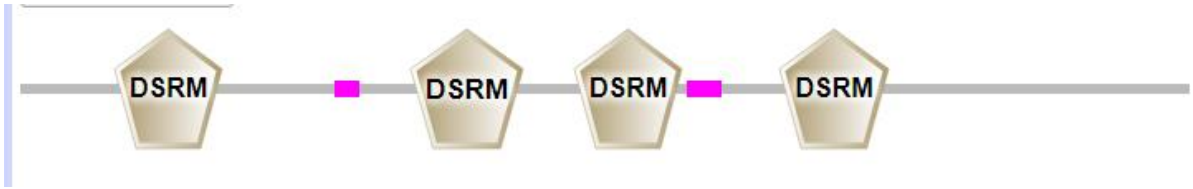

Leptinotarsadecemlineata XP_023023346.1

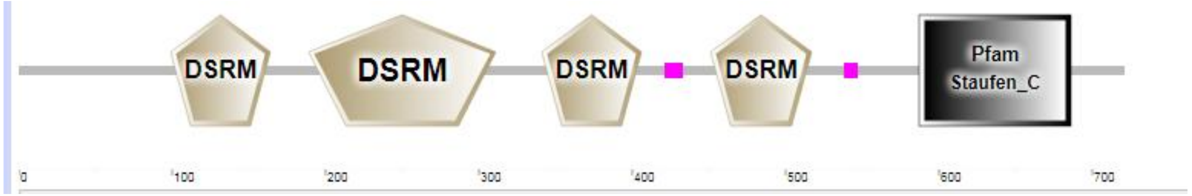

*Triboliumcastaneum*EFA11564.2

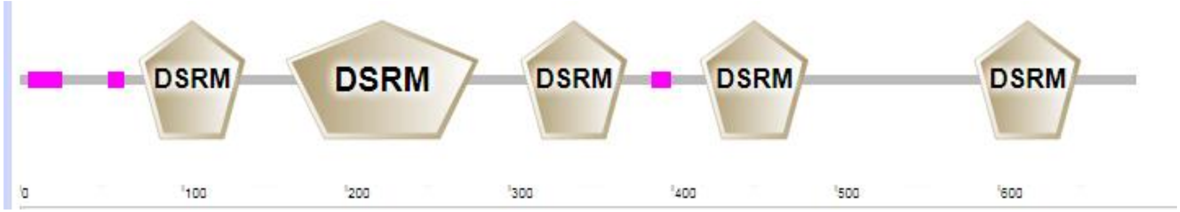

Onthophagustaurus XP_022914722.1

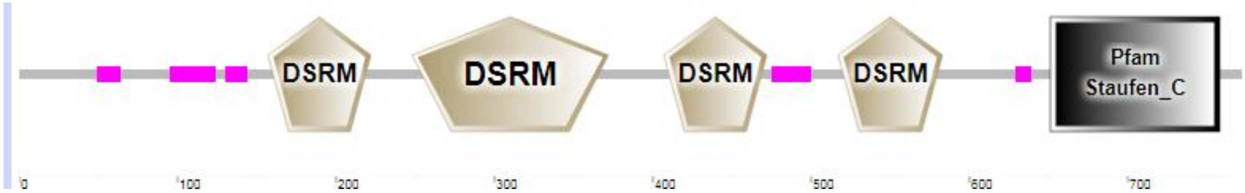

Riptortuspedestris BAN21133.1

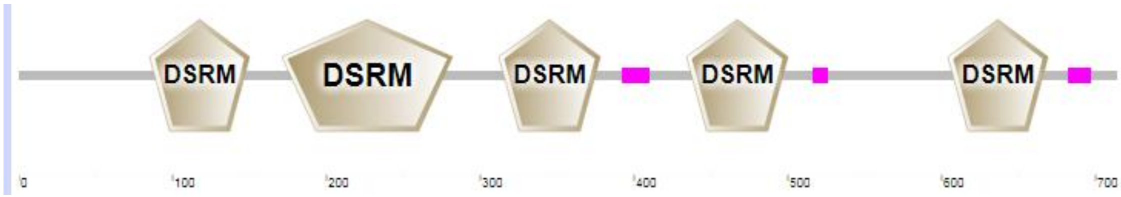

*Culexquinquefasciatus*XM_001841817.1

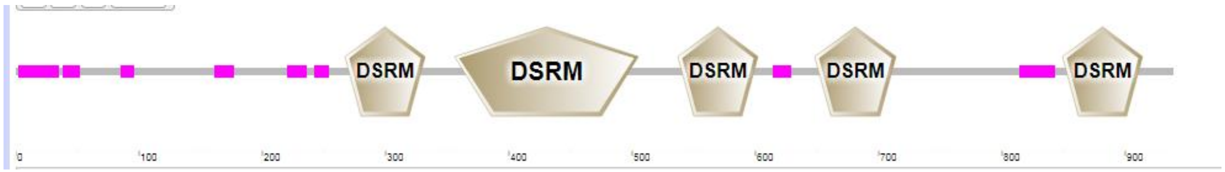

Phenococcussolenopsis (SRP133470)

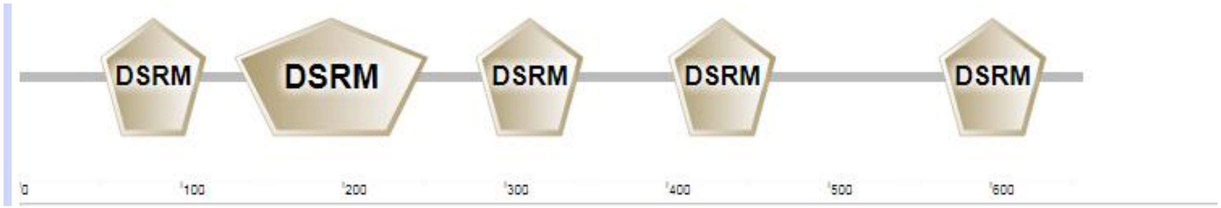

Bemisiatabaci(MAMS00000000)

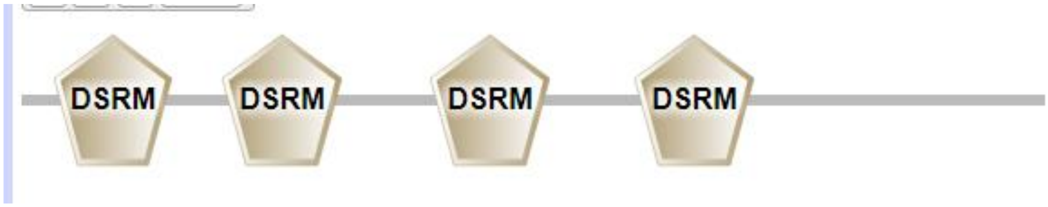

*Cimexlectularis*XM_014397141.2

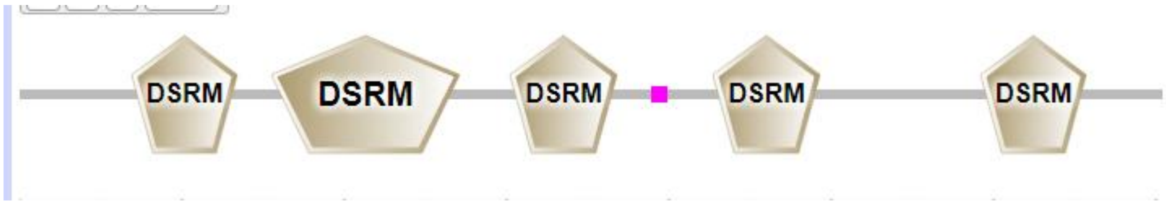

*Drosophila malenogaster*NM_001169714.2

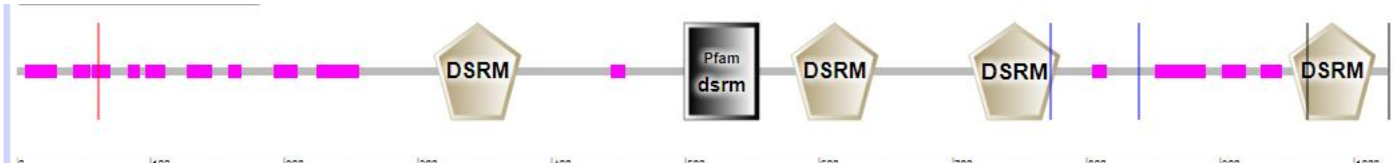

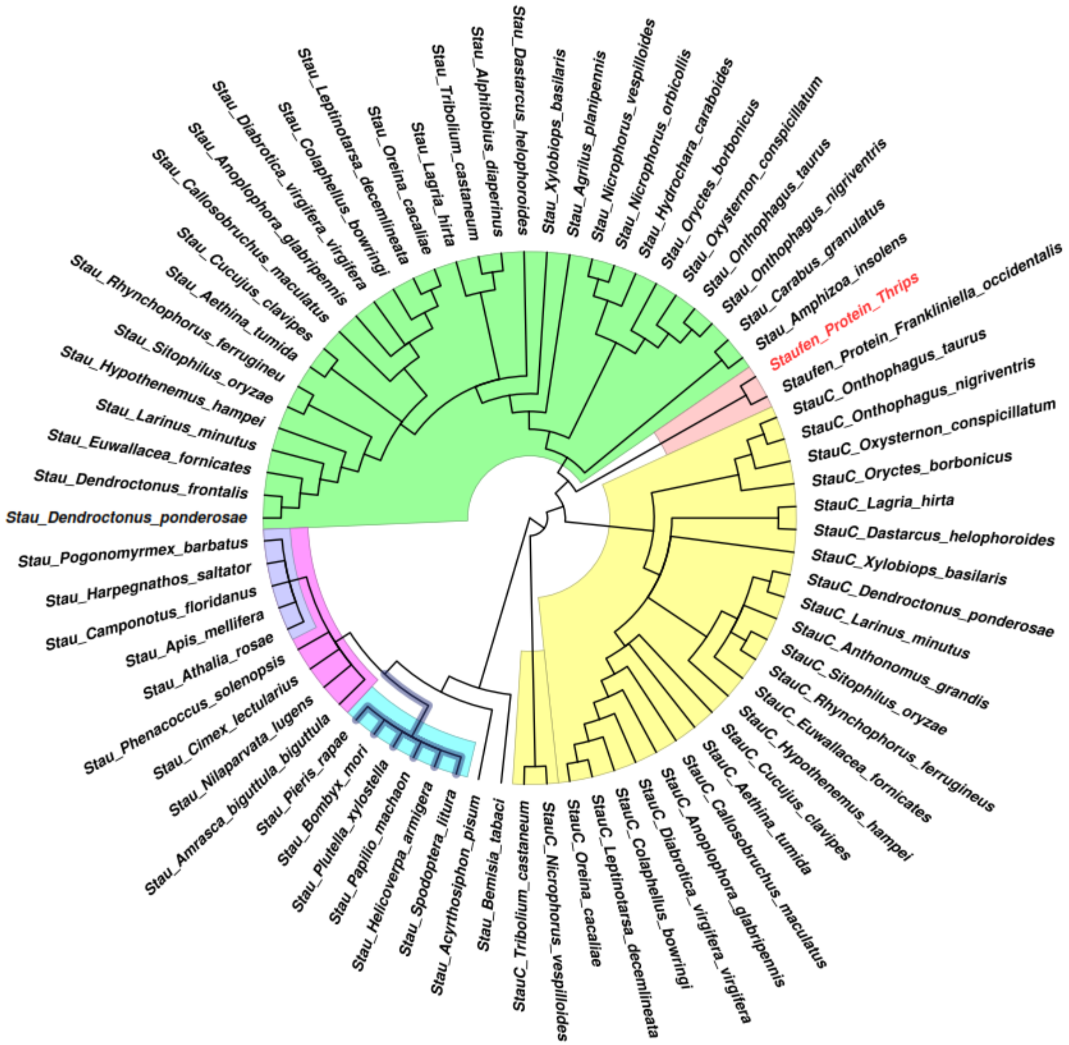

Fig. S2 Phylogenetic analysis of Staufenand StauCsequences within insects of different orders

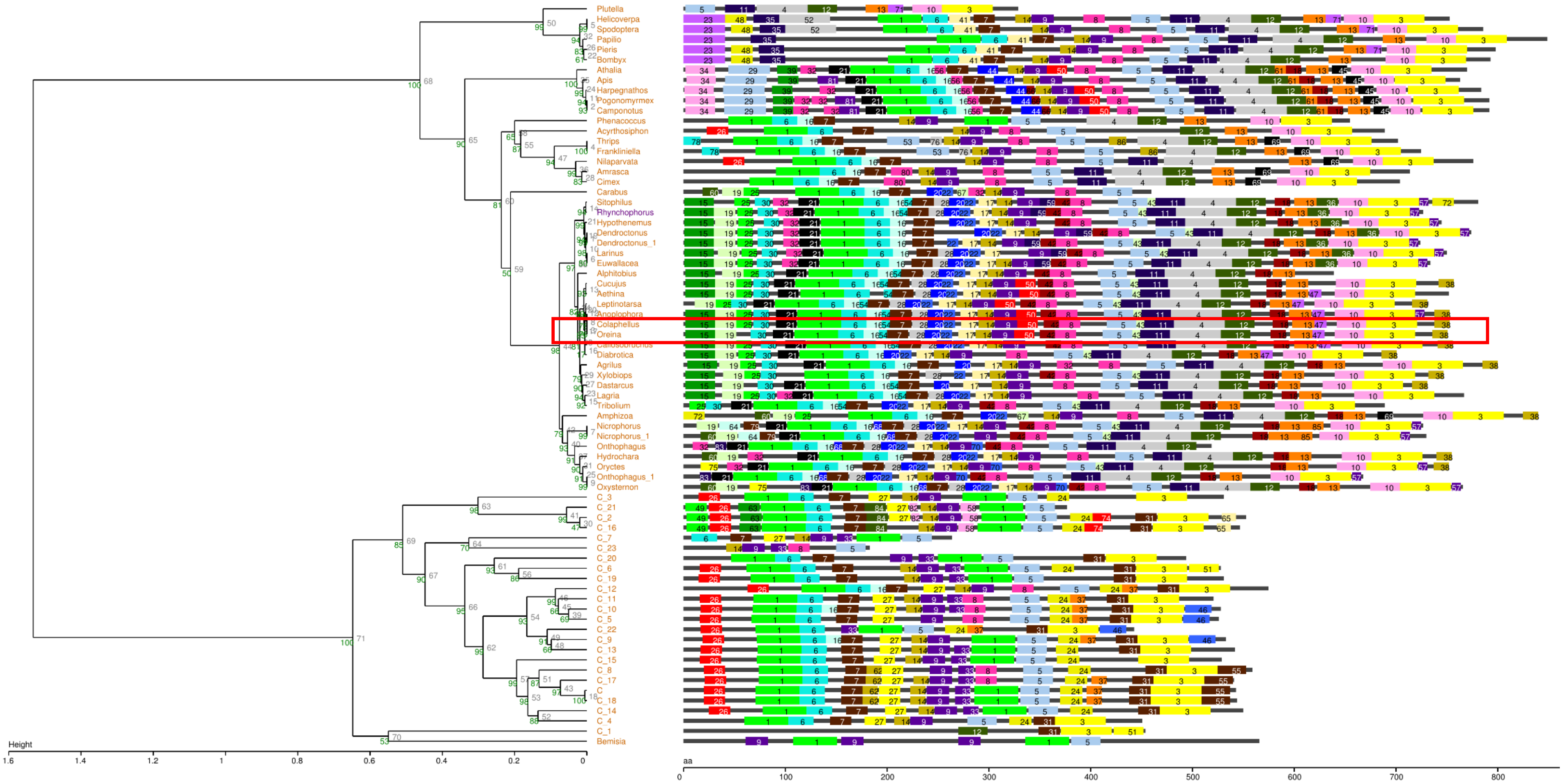

Unique Motifs

Motif 78: >Thrips- MPAPVMVQSAPAQPSGF>Frankliniella-MPSPVMVQSAPAQPSGY

Motif 53:>Thrips-YFRPAYPGQVRPSNPCGEMAAWGHPRPPMNMY >Frankliniella-YNHHQVEFQIRPPNPCGEMAAWGPPRPTINMY

Motif 76:>Thrips-QRYYAKPSIYP>Frankliniella-QRYYAKPSIYP

Motif 86:>Thrips-NLIKEPKPASPVESPTH>Frankliniella-NLIKEPKPASPVESPTH

Fig.S3 Phylogenetic analysis of Staufen and StauC sequences within insects of different orders using SALAD for the depiction of unique protein motifs in *Thripstabaci*
