# Supplementary material for: RNA sequencing, selection of reference genes and demonstration of feeding RNAi in Thrips tabaci (Lind.) (Thysanoptera: Thripidae)
Source: BMC Mol Biol. 2019 Feb 18;20:6. doi: 10.1186/s12867-019-0123-1 (PMC6380046; doi:10.1186/s12867-019-0123-1)
Supplement: Supplementary file 6 — Additional file 6. RNA quality assessment for transcriptome sequencing and dsRNA feeding schematic layout. [file 12867_2019_123_MOESM6_ESM.pdf]

Fig. S1 Quality of RNA samples on 1% denatured Agarose gel (SI denotes RNA of *Thrips tabaci*; BI sample is not related to this manuscript)

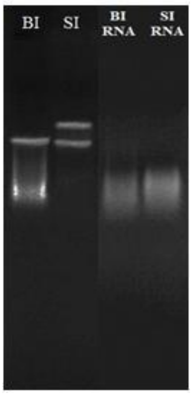

Fig. S2 Library Profile of Thrips RNA on Agilent Tape Station using High sensitivity D1000 Screen Tape

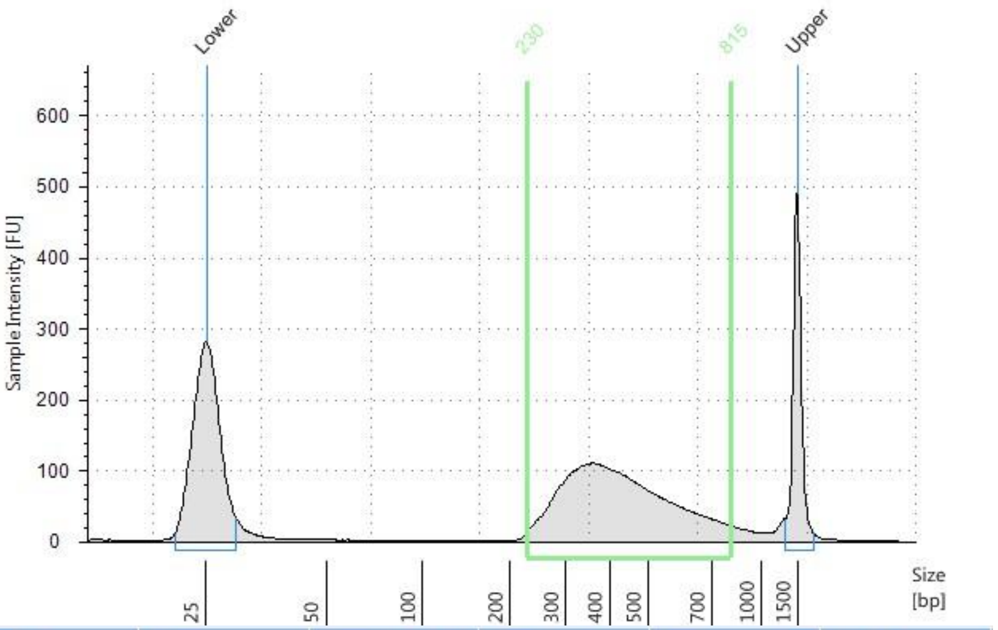

From [bp]	To [bp]	Average Size [bp]	Conc. [pg/µl]	Region Molarity [pm	% of Total
230	815	436	702	2700	86.88

Fig. S3 Feeding RNAi assay setup for *Thrips tabaci*

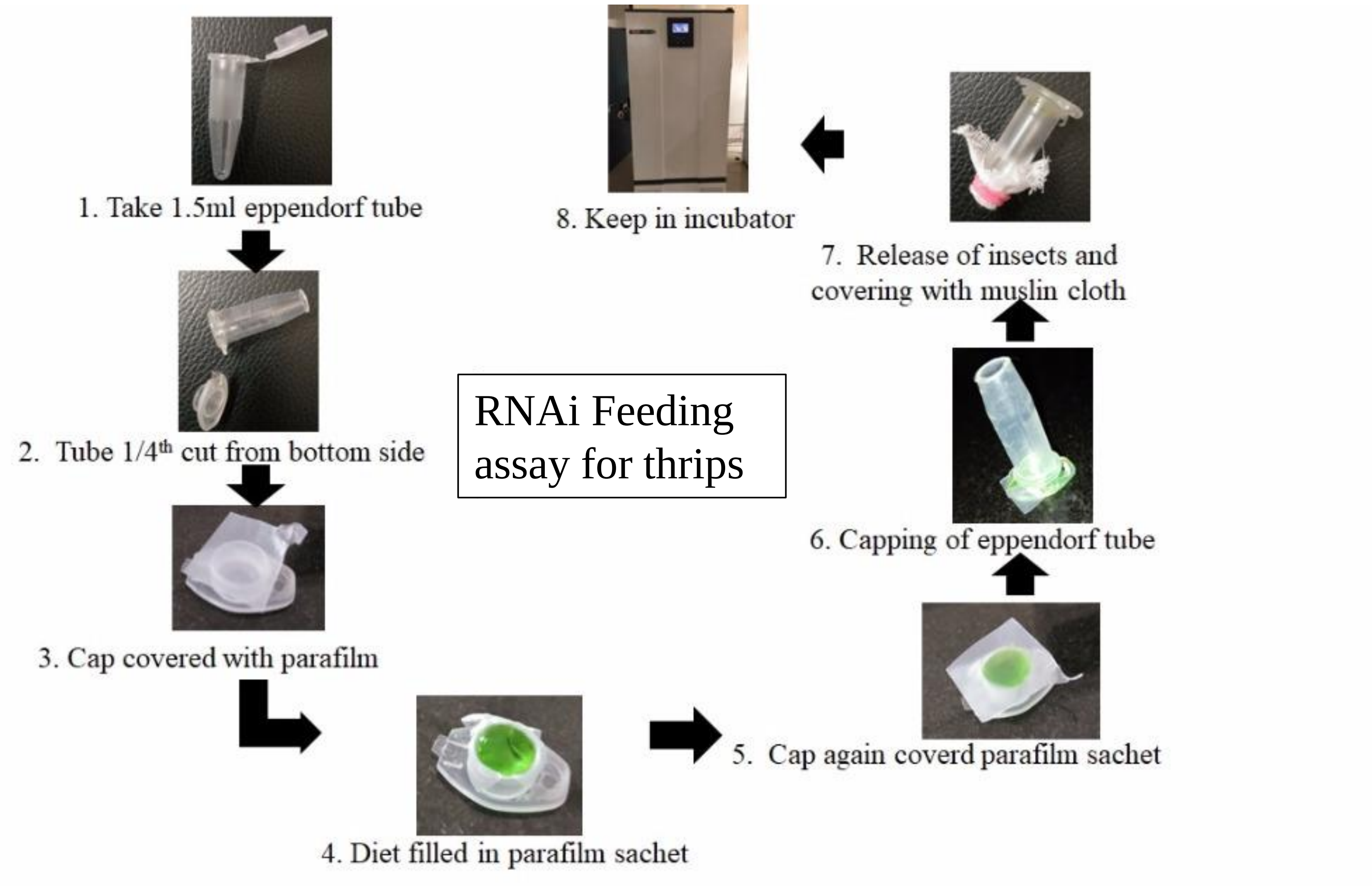

Modified after Singh et al 2018 (dx.doi.org/10.17504/protocols.io.ksgcwbw)
